# Foodborne and neglected parasitic zoonoses in Ethiopian red meat animals: insights from a systematic review and meta-analysis

**DOI:** 10.3389/fvets.2025.1501940

**Published:** 2025-06-04

**Authors:** Melkie Dagnaw Fenta, Biruk Eshetu, Abebe Belete Bitew, Haileyesus Dejene, Mebrie Zemene Kinde

**Affiliations:** ^1^Department of Clinical Veterinary Medicine, College of Veterinary Medicine and Animal Science, University of Gondar, Gondar, Ethiopia; ^2^Department of Parasitology, College of Veterinary Medicine and Animal Science, University of Gondar, Gondar, Ethiopia; ^3^Department of Veterinary Epidemiology and Public Health, College of Veterinary Medicine and Animal Science, University of Gondar, Gondar, Ethiopia; ^4^Department of Veterinary Biomedical Science, College of Veterinary Medicine and Animal Science, University of Gondar, Gondar, Ethiopia

**Keywords:** Ethiopia, foodborne, parasite, pooled prevalence, zoonosis

## Abstract

**Background:**

The most prevalent yet neglected cestode meat-borne parasitic zoonoses are bovine cysticercosis and cystic echinococcosis, while the most common meat-borne protozoan zoonoses are toxoplasmosis and cryptosporidiosis in Ethiopia. In Ethiopia, bovine cysticercosis, cystic echinococcosis, toxoplasmosis, and cryptosporidiosis are the most common but neglected meat-borne parasites. The main transmission route is through contaminated meat products. The aim of this review was to provide an overall prevalence estimation of major food-borne zoonotic parasitic in ruminants in Ethiopia.

**Methods:**

The present meta-analysis was conducted based on the Preferred Reporting Items for Systematic Reviews and Meta-Analyses (PRISMA) guidelines. Articles were searched in PubMed, Google Scholar, Web of Science, and HINARI. A total of 104 eligible articles were included in the final meta-analysis. The pooled prevalence estimates and 95% confidence intervals (CI) were conducted using random effect model, and heterogeneity was assessed using I^2^ statistics.

**Results:**

Toxoplasmosis had the highest pooled prevalence (38, 95% CI: 30–46%), followed by cystic echinococcosis (25, 95% CI: 18–32%), cryptosporidiosis (14, 95% CI: 9–19%), and bovine cysticercosis (9, 95% CI: 5–13%). In most individual analyses, Egger’s regression test did not reveal significant publication bias, since the *p*-values were greater than 0.05. Regional subanalysis showed that bovine cysticercosis was most prevalent in the Amhara region (16, 95% CI: 6–13%), whereas cystic echinococcosis was highest in Oromia (33, 95% CI: 22–45%) and Tigray (29, 95% CI: 24–33%). Host-wise analysis indicated that toxoplasmosis was most prevalent in sheep (41%), followed by goats (39%), and cattle (28%). Cryptosporidiosis was most commonly detected in cattle (16%), sheep (11%), and goat (8%). Age-based analysis revealed a higher prevalence in calves and lambs with an estimated pooled prevalence of 15% (*I*^2^ = 83%).

**Conclusion:**

The increasing prevalence of meat-borne parasitic zoonoses in Ethiopia highlights the need for urgent intervention. Strengthening disease surveillance, enforcing strict meat inspection protocols, and raising public awareness about zoonotic risks are critical for effective control. A coordinated approach between veterinary professionals, public health authorities, and policymakers is essential to mitigate the burden of these neglected parasitic infections and protect both animal and human health.

## Introduction

Ethiopia has the largest livestock population and the second largest human population in Africa and has experienced a substantial contribution from its livestock sector to the country’s economy, which also holds promise for further economic development (1). Among the diverse livestock in Ethiopia, cattle and small ruminants hold the utmost importance, with populations of 70 million cattle, 52.5 million sheep, and 42.9 million goats (2).

In developing countries, livestock farming plays a crucial role in ensuring food security; however, it also entails zoonotic risks (3). Zoonosis, a public health concern, has been recognized in numerous developing nations and encompasses a wide range of aetiologies, including parasitic, viral, bacterial, rickettsia, and chlamydial zoonoses. Zoonotic diseases account for a substantial proportion, specifically 60.3%, of infectious diseases (4). Animal-derived foods, such as meat, milk, and eggs, are globally acknowledged as fundamental components of the human diet (5). Nonetheless, the consumption of contaminated animal-based foods poses a significant threat to human health. Mammals, particularly bovine, ovine, and caprine species, serve as the primary sources of zoonotic diseases transmitted through food (6).

Among the health challenges faced, parasitic infections pose a significant burden, resulting in 450 million illnesses and 200,000 deaths annually (7). Among the 1,500 infectious agents identified in humans, 66 are protozoa, and 287 are helminths (8). Currently, food-borne zoonotic parasitic diseases have a global impact on food safety and human health because of the consumption of contaminated food, particularly animal products. These agents significantly affect public health and the economic sector (9).

Parasitic zoonotic diseases encompass a diverse group of infectious diseases with varying host ranges and transmission patterns, and their distribution, prevalence, and transmission patterns are influenced by factors related to humans, animals, and the environment (10). Ethiopia bears a substantial burden of zoonotic diseases, exacerbated by the lack of coordination between the human and animal health sectors and inadequate resources for public health systems. These factors contribute to weak surveillance systems and inefficient responses to public health threats within the country. Specifically, the issue of food-borne parasitic zoonosis in Ethiopia is further complicated by the absence of effective inspection at critical control points in slaughterhouses, limited awareness and knowledge regarding the mode of transmission, and the widespread practice of consuming raw meat in both rural and urban communities (11). Poor hygiene regulations, inadequate food safety laws, and lack of training for food processors contribute significantly to the issue (12). The National Hygiene and Sanitation Strategy Program attribute around 60% of Ethiopia’s disease burden to poor hygiene and sanitation. The absence of strict slaughter regulations and widespread backyard slaughtering worsens the prevalence of meat-borne parasitic zoonoses. Private slaughtering, especially during traditional celebrations, and practices like Kircha, where merchants prioritize profit over hygiene, lead to the consumption of contaminated meat. Several parasitic zoonoses, including bovine cysticercosis, toxoplasmosis, cryptosporidiosis, and echinococcosis, are prevalent in Ethiopia.

Bovine cysticercosis and cystic echinococcosis are major zoonotic diseases with both economic and public health impacts (13, 14). Bovine cysticercosis, caused by the cystic stage of *Taenia saginata*, affects human health through the consumption of raw or undercooked meat from infected cattle (15). Intestinal taeniosis, linked to this infection, can cause symptoms like abdominal discomfort, intestinal irritation, obstruction, nausea, weight loss, and anal pruritus (16). The disease also leads to financial losses due to carcass condemnation in the dairy industry. Its spread is influenced by cattle rearing practices, meat inspection, and the consumption of raw meat, while poor hygiene and lack of awareness contribute to transmission. Globally, there are about 77 million carriers of *Taenia saginata*, with 40% in Africa (17). The prevalence of bovine cysticercosis in cattle in Ethiopia varies from 3.1% in the central region to 26.25% in the southern region of the country (18–20). *Cystic echinococcosis* is a zoonotic parasitic disease that is induced by the cystic form of *Echinococcus. granulosus* and has considerable public health and economic importance. *Echinococcus. granulosus* is primarily perpetuated in a domestic cycle involving the dog (*Canis familiaris*) as the definitive host and domestic ungulates (sheep, cattle) as intermediate hosts (21).

A tapeworm, which is recognized for inhabiting the small intestine of its definitive host, is also found in its larval stage within various organs and tissues of intermediate hosts, including the liver, lungs, brain, muscles, lymph nodes, and other regions (22). This disease is more prevalent in developing countries, especially in rural communities where dogs live in close proximity to humans and domestic herbivores. The highest incidence is reported in regions where sheep and cattle are raised (23). Different regions across the country frequently report human hydatidosis, in which a close relationship exists between dogs and domestic animals in Ethiopia (24). In cattle, studies conducted at various abattoirs across the country have revealed that the prevalence of hydatidosis ranges from 9.4 to 63.7% in Harar and Assella, respectively. This disease plays a significant role in diminishing both the quantity and quality of exported goods. The consequences of this infection in humans and intermediate hosts include the formation of hydatid cysts in the lungs, liver, or other organs (25). While the occurrence of this clinical disease in domestic animals is rare, it poses a greater threat to humans. As reservoirs, domestic animals hold immense importance as hosts for this parasite (22). As the cysts gradually grow in size, they can detrimentally impact the well-being of the host. When located in the lungs, they result in dyspnoea, whereas in the liver, they cause digestive disturbances and ascites (26).

Toxoplasmosis is caused by the intracellular parasite Toxoplasma gondii (*T. gondii*), which spreads globally through meat transmission (27). Domestic cats serve as the definitive host, while warm-blooded animals and humans act as intermediate hosts. Infection occurs via the consumption of raw or undercooked meat from infected animals like sheep, pigs, and cattle. While generally opportunistic in healthy individuals, *T. gond*ii poses serious risks to those with weakened immune systems, such as HIV/AIDS patients, pregnant women, cancer patients, or organ transplant recipients (28, 29). The prevalence in sheep is about 30%, with active infections estimated at 15% globally. In goats, prevalence can reach up to 77%, varying by region (30). In Ethiopia, various well-documented reports on toxoplasmosis exist. The research conducted an extensive serological survey, revealing the presence of *T. gondii* infection in sheep and goats, with a prevalence of 43% (31). Additionally, a serological survey performed by Teshale et al. (32) revealed the prevalence of caprine toxoplasmosis in Central and Southern Ethiopia, revealing a remarkable prevalence of 74.8%. In a recent study, a seroprevalence of 70% for toxoplasmosis was reported in goats and sheep in the northwest region of Ethiopia (33).

Toxoplasmosis is considered a disease of significant economic importance because of its ability to cause notable harm through reproductive complications, inflammation of the nervous system, and respiratory infections, particularly in sheep and goats (34). Cryptosporidiosis is a severe parasitic disease caused by protozoan parasites belonging to the *Cryptosporidium* genus (35). These parasites can cultivate and proliferate within the brush border of gastrointestinal cells in infected animals and humans, leading to severe health issues (36). The genus *Cryptosporidium* comprises 13 recognized species distinguished on the basis of morphological characteristics, host specificity, and DNA-oriented investigations (37). However, only two species, *Cryptosporidium parvum* and *Cryptosporidium andersoni*, are significant in the context of cattle (38). The primary modes of transmission for this zoonotic disease are through contaminated water, contaminated food, and contact with infected animals (39, 40). Calves serve as reservoirs for the zoonotic species *Cryptosporidium parvum* (*C. parvum*), which is responsible for causing diarrhea in humans on a global scale. A recent survey conducted in the United Kingdom revealed that 38.5% of cryptosporidiosis cases in humans were attributed to *C. parvum*, with 25% of these cases occurring through direct contact.

The occurrence of *Cryptosporidium* infection in cattle can be associated with factors such as age, bedding depth, and environmental cleanliness (41). This parasite follows a homoxenous life cycle and has a significant capacity to reproduce and spread among various animal hosts (18). Reports have indicated that the prevalence of cryptosporidiosis in cattle ranges from 6.25 to 39.65% in different regions of the world. The disease is characterized by symptoms such as loss of appetite, diarrhea, stunted growth, and mortality (42, 43). The increasing human population and socioeconomic shifts have resulted in the migration of people into new ecological niches and changes in animal husbandry practices, thereby significantly impacting the emergence and burden of disease (44). Furthermore, both natural and human-induced environmental changes and disturbances continue to have substantial effects on the prevalence and emergence of zoonotic parasitic diseases. The spread and epidemiology of parasitic zoonoses are significantly influenced by human behavior. Unlike viruses and bacteria, parasites possess unique characteristics that enable them to thrive in the environment and be transmitted by animals and animal products. Some have the potential to cause significant public health problems, whereas others can cause substantial losses in livestock, leading to significant economic and production losses.

This systematic review aims to assist professionals in animal, human, and environmental health in monitoring foodborne parasitic zoonotic diseases. It serves as a resource for developing effective control and prevention strategies and enhancing epidemiological surveillance systems. Additionally, it supports the one health approach and raises awareness about neglected zoonotic diseases in animal-derived food in Ethiopia. The objective was to estimate the prevalence of common foodborne parasitic zoonosis (bovine cysticercosis, toxoplasmosis, cryptosporidiosis, and cystic echinococcosis) in domestic ruminants in Ethiopia, addressing specific research questions.

What is the prevailing frequency of specified parasitic zoonosis contracted through food of ruminant animal origin?

Which specific organ harbors foodborne parasitic zoonosis?

Which region of Ethiopia is more prone to foodborne parasitic zoonosis?

Which foodborne parasite zoonosis is highly distributed in Ethiopia?

## Methods

A systematic assessment of published studies reporting the prevalence of toxoplasmosis, cryptosporidiosis, bovine cysticercosis and cystic echinococcosis among different food animal species (Bovine, Ovine and Caprine) was performed based on PRISMA checklist (45) (Supplementary material 1).

### Study eligibility criteria

#### Inclusion criteria

This meta-analysis included primary studies published in English that reported the prevalence of bovine cysticercosis, cystic echinococcosis, toxoplasmosis, and cryptosporidiosis in domestic ruminants (cattle, sheep, and goats) or related food sources. Eligible studies provided a clear estimation of the proportion of these foodborne parasitic zoonoses. Observational studies assessing prevalence were considered, provided the animals were not experimentally infected. Parasite identification was required at least to the genus level, and studies had to be conducted in Ethiopia.

#### Exclusion criteria

Studies on parasitic zoonoses in pigs, camels, or other non-ruminant species were excluded. Additionally, studies lacking clear prevalence estimates for each parasite–host relationship were omitted. Excluded materials also included review articles, duplicates, and abstracts with insufficient data, qualitative studies, KAP-based studies, book chapters, case reports, editorials, short communications and opinions. Intervention studies without baseline data on animal exposure and disease association were also excluded.

#### Information sources

The literature search was conducted from October 2022 to January 2024. The database sources PubMed, HINARI, Web of Science, Google Scholar and other manual open internet methods were used. The included studies were reported from Ethiopia. It is based on the reported prevalence of foodborne parasitic zoonosis, mainly in slaughterhouses and other study areas (farms with selected parasites).

#### Search strategy

We selected the most widespread and public health-relevant foodborne parasitic zoonoses of animal origin in Ethiopia. To ensure a systematic and unbiased approach, two authors (MD and MZ) independently developed a comprehensive search strategy, with any disagreements resolved through discussion with a third author (AB). The search was guided by the CoCoPop (Condition, Context, and Population) framework, where the condition focused on foodborne parasitic zoonoses, the context was Ethiopia, and the population included domestic ruminants such as sheep, goats, and cattle. To enhance the transition between the search strategy and data extraction, we carefully structured the search process using Boolean operators (“AND/OR”) to refine and expand the search results by combining related terms. In PubMed, the search was conducted using the “All Fields” function, employing a query that included keywords related to prevalence, epidemiology, parasitic zoonoses, and specific infections such as toxoplasmosis, cysticercosis, cystic echinococcosis, and cryptosporidiosis. The search was further filtered by animal species, including cattle, sheep, and goats, and restricted to studies conducted in Ethiopia.

In the Web of Science, a broader approach was applied by searching within the “Topic Field,” which includes the title, abstract, and author keywords. The search terms incorporated variations of parasite infections, foodborne transmission, epidemiology, prevalence, and infection rates. Additionally, specific geographic identifiers such as Ethiopia and its regional states (Addis Ababa, Afar, Amhara, Oromia, SNNPR, Gambella, Harari, Somali, and Tigray) were included to ensure comprehensive coverage of relevant studies.

### Data extraction

After screening studies based on the eligibility criteria, two investigators (MD and BE) independently extracted relevant data. Any discrepancies were resolved through discussion with a third author. The extracted data encompassed both quantitative and qualitative information and were systematically organized into Word tables and an Excel spreadsheet. The final dataset comprised 104 articles, including details on: Primary author’s name, Publication and study year, Geographical location, Study animal species (host), Sample size and number of positives, diagnostic methods, data collection methodologies, Ethical considerations, prevalence and principal findings of the parasite type.

### Study quality assessment

In this review, the quality of the included studies was evaluated using the AMSTAR-2 tool (Supplementary material 2), as the methodological rigor of the studies directly influences the reliability of the meta-analysis results. Two investigators (MD and AB) independently assessed study quality, applying the 15-item AMSTAR-2 checklist, which covers both randomized and nonrandomized trials of health interventions. Some items are applicable to both types of studies, while others are specific to either randomize or nonrandomized trials. Since this meta-analysis focused on nonrandomized (observational) studies, only the relevant items were used. The quality assessment is integral in determining the confidence in the results and ensuring that the conclusions drawn from the meta-analysis are based on studies with sound methodologies. Regarding data extraction, two investigators (MD and BE) independently extracted relevant data from the selected studies. In the case of any discrepancies, the investigators resolved disagreements through discussion and consultation with a third author (AB), ensuring consensus and consistency in the data.

### Data synthesis and statistical analysis

The random effects model, which employs the restricted maximum likelihood method (REML) to compute within- and between-study variability, was employed to estimate the aggregated prevalence and 95% confidence intervals. This model was utilized to conduct a comprehensive meta-analysis (overall effect size (ES) or often represented as a percentage or proportion), assess heterogeneity, and determine the weight of each study. Furthermore, graphs and tables were employed to illustrate the prevalence status of the primary parasitic zoonosis on the basis of the geographical distribution of animal species, the type of parasites, and the study years.

To estimate the prevalence, we extracted data from the number of events and the total number of samples. These data were then subjected to proportional meta-analysis via the “metaprop” function of the “meta” package version 4.1.3-0 in Balduzzi et al. (46) R statistical software. Pooled proportions of prevalence were estimated via logit transformation via a logistic-normal random effects regression model. Subgroup analysis was conducted via the mixed effect logistic regression model.

### Investigation of heterogeneity

The Cochran’s Q test (indicated by the *p* value), τ2 (representing between-study variance), and the inverse variance index (I^2^) were employed to assess the sources of heterogeneity. The I^2^ index, as elucidated by Higgins and Thompson (47), was calculated to indicate low, moderate, and high levels of heterogeneity, corresponding to *I*^2^ values of 25, 50, and 75%, respectively. Heterogeneity was deemed statistically significant if the I^2^ value exceeded 50%, and the Q test yielded a *p* value less than 0.10. τ^2^ (tau-squared) measures the variance between studies. In simple terms, it tells us how much the true effects differ from study to study. I^2^ (I-squared) indicates the percentage of variability across studies that is due to heterogeneity rather than chance. For example, if *I*^2^ = 50%, this means that 50% of the variation between study results is due to true differences rather than random sampling error. By using these indices, we were able to identify the sources and extent of heterogeneity in the meta-analysis.

The extent of study heterogeneity was evaluated through a forest plot diagram, which depicted the weights, effect magnitudes, and 95% confidence intervals for between-study variability. A subgroup analysis of the prevalence of the primary prevalence of toxoplasmosis, cryptosporidiosis, bovine cysticercosis and cystic echinococcosis was performed, considering factors such as publication year, study location or region, species of study animals, and methods of detection.

### Publication bias assessment

To assess publication bias, funnel plot diagrams and Egger’s regression test were used. A funnel plot is a scatter plot of the effect sizes from individual studies against their sample sizes. If there is no publication bias, the plot should resemble a symmetrical funnel. Egger’s regression test is a statistical method that helps determine whether the funnel plot is symmetrical. If the plot is asymmetrical (i.e., the studies are skewed in one direction), it suggests potential publication bias, meaning that smaller or less significant studies may be missing from the analysis. A *p*-value from the test greater than 0.05 typically indicates that the funnel plot is symmetric, suggesting no significant publication bias.

## Results

### Literature search results

**Figure 1:**
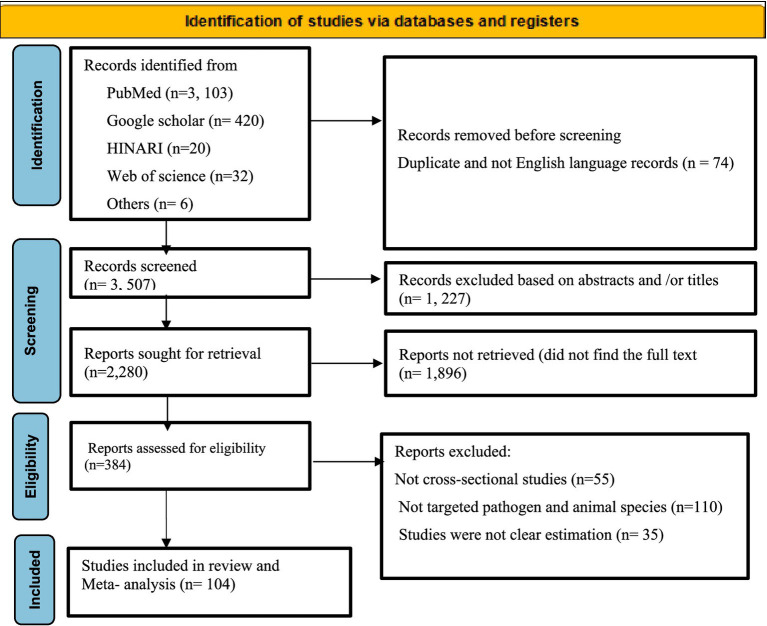
Flow chart of selected studies on foodborne parasitic zoonotic diseases of animal origin.

According to the PRISM 2020 flow chart (Figure 1), an initial search identified 3,581 articles across various electronic databases. After excluding 74 articles due to duplicates and non-English records, 3,507 articles remained. Of these, 1,227 were eliminated based on a review of titles and abstracts. A total of 2,280 articles were then sought for retrieval, but 1,896 could not be obtained. Out of the 384 articles assessed for eligibility, 200 were excluded for various reasons. Ultimately, 104 studies were included in the meta-analysis.

### Overview of the included articles in the meta-analysis

This systematic review included 104 studies focusing on four major zoonotic parasitic diseases affecting domestic ruminants in Ethiopia: bovine cysticercosis, cystic echinococcosis, toxoplasmosis, and cryptosporidiosis. All of the investigations employed an observational study design.

### Bovine cysticercosis

The current systematic review includes a total of 34 articles on cysticercosis in cattle, with each article presenting one or multiple examinations of parasites (14). These articles were published between 2008 and 2023. Notably, the individual studies included in this systematic review reported a minimum sample size of 234 in Northeast Ethiopia (48) and a maximum sample size of 4,456 (49) in the Amhara Region in Northwest Ethiopia. The overall number of animals sampled throughout the years of the study was 25,065, of which 3,076 tested positive for bovine cysticercosis. The majority of the data collection methods utilized by observations conducted at abattoirs and surveys conducted through questionnaires. A comprehensive overview of the investigated studies is provided in Table 1.

**Table 1:** Characteristics of the studies of *Cysticercos bovis* (*Taenia saginata* cysticercos) (*n* = 34).

First author	Study year	G. Location	Regions	ST	Methods diagnosis	Data collection	Total examine	Positive	Prevalence
Tolosa et al. (80)	2007–2008	Southwestern	Oromia	SR	AM and PM	Abattoir survey and questionnaire	512	15	0.0293
Regassa et al. (81)	2007–2008	Southern	Oromia	SR	AM and PM	Abattoir survey and questionnaire	415	47	0.113
Terefe et al. (82)	2009–2010	Southern	SNNPR	SYR	AM and PM	Abattoir survey and questionnaire	898	177	0.197
Ibrahim and Zerihun (83)	2010–2011	Central	AA	SYR	AM and PM	Abattoir survey	535	19	0.036
Emiru et al. (84)	2013–2014	Central	Oromia	SYR	AM and PM	Abattoir survey	430	24	0.056
Hirpha et al. (85)	2015–2016	Southern	SNNPR	SR	AM and PM	Mixed (abattoir, questionnaire, and drug sale inventory)	384	33	0.086
Edao et al. (86)	2014–2015	Southeast	Oromia	SR	AM and PM	Abattoir survey	430	5	0.012
Megeresa et al. (87)	2008–2009	Southwest	Oromia	SR	PM	Abattoir survey	500	22	0.044
Abunna et al. (14)	2005–2006	Southern	SNNPR	SR	PM	Abattoir survey, interview, questionnaire	400	105	0.263
Bedore and Geinero (88)	2017–2018	Central	AA	SYR	PM	Abattoir survey	317	29	0.091
Moje et al. (89)	2012–2013	Southwest	Oromia	SYR	AM and PM	Abattoir survey	405	203	0.5
Sheferaw and Abdu (1)	2015–2016	Southwest	Oromia	SYR	PM	Abattoir survey and questionnaire	1,200	80	0.067
Tamirat et al. (11)	2017–2018	Northwest	Amhara	SYR	AM and PM	Abattoir survey and questionnaire	480	20	0.042
Engdaw et al. (90)	2009–2010	Northeast	Amhara	SR	PM	Abattoir survey	421	27	0.064
Mesele et al. (91)	2011–2012	Northwest	Amhara	SR	AM and PM	Abattoir survey	532	92	0.173
Tegegne et al. (48)	2016–2017	Northeast	Amhara	SR	AM and PM	Abattoir survey and questionnaire	234	21	0.09
Yigzaw et al. (92)	2015–2016	Northeast	Amhara	SR	AM and PM	Mixed (abattoir, questionnaire, drug sale inventory)	384	26	0.068
Bedu et al. (93)	2010–2011	Southern	Oromia	SR	PM	Mixed (abattoir, questionnaire, drug sale inventory)	400	96	0.24
Kassaw et al. (94)	2015–2016	Northeast	Amhara	SR	AM and PM	Abattoir survey	425	20	0.047
Tesfaye et al. (95)	2010–2011	Southern	SNNPR	SR	AM and PM	Abattoir, questionnaire, and drug sale inventory	540	14	0.026
Teklemariam and Debash (96)	2014–2015	Central	AA	SR	AM and PM	Mixed (abattoir, questionnaire, drug sale inventory)	384	10	0.026
Abay and Kumar (97)	2007–2008	Northeast	Tigray	SR	AM and PM	Abattoir survey	1,023	74	0.072
Abunna et al. (14)	2011–2012	Southern	SNNPR	SR	PM	Abattoir, questionnaire, and drug sale inventory	400	48	0.12
Tadesse et al. (98)	2005–2006	Southwest	Oromia	SYR	AM and PM	Abattoir, questionnaire, and drug sale inventory	1,216	56	0.046
Abera et al. (99)	2020–2021	Southwest	Oromia	SYR	AM and PM	Abattoir, questionnaire, and drug sale inventory	1,108	302	0.273
Tesfaye et al. (100)	2013–2014	Southern	SNNPR	SYR	AM and PM	Abattoir survey	400	17	0.043
Bayou and Taddesse (101)	2016–2017	Southwest	Oromia	SR	AM and PM	Abattoir survey	384	25	0.065
Bekele et al. (102)	2016–2017	Southwest	Oromia	SR	AM and PM	Mixedabattoir, questionnaire	600	93	0.155
Netsanet et al. (75)	2018–2019	Northeast	Amhara	SR	AM and PM	Mixedabattoir, questionnaire	384	37	0.096
Kumar et al. (103)	2005–2006	Northeast	Tigray	SR	PM	Abattoir survey	3,711	308	0.083
Fesseha and Asefa (104)	2021–2022	Central	AA	MRS	AM and PM	Abattoir survey, quantitative	330	14	0.042
Mussa (105)	2021–2022	Southern	SNNPR	SR	PM	Abattoir survey	422	152	0.36
Kebede (49)	2006–2007	Northwest	Amhara	SYR	PM	Abattoir survey	4,456	824	0.1849
Moje et al. (89)	2011–2012	Southern	Oromia	SR	AM and PM	Mixed (abattoir, questionnaire, drug sale inventory)	405	41	0.101

AM, Ante mortem; PM, post-mortem; G. location, Geographical location; SYR, Systematic random; SR, Simple random; MRS, Multistage random sampling; SNNPR, Southern nation and nationalities of peoples region.

### Cystic echinococcosis

This systematic review provides a comprehensive analysis of 37 studies on cystic echinococcosis in domestic ruminants, including cattle, sheep, and goats. In this systematic review, the largest sample size examined in a study was 5,194 animals, whereas the smallest study analyzed only 246 animals. A total of 28,024 animals were examined and among them, 7,277 were positive for cystic echinococcosis. Table 2 provides a comprehensive summary of the reviewed studies.

**Table 2:** Characteristics of the studies included the prevalence of cystic echinococcosis in Ethiopia (*n* = 37).

First author	Publication year	Study year	Location	Region	Host	Study design	Total examine	Positive	Prevalence
Tolosa et al. (80)	2009	2007–2008	Southwestern	Oromia	Cattle	CS	512	161	0.314
Regassa et al. (81)	2009	2007–2008	Southern	SNNPR	Cattle	CS	415	64	0.154
Bayu et al. (106)	2012	2011–2012	Central	AA	Sheep, goat	CS	1,152	31	0.027
Belina et al. (107)	2015	2011–2012	Southwestern	Oromia	Cattle	CS and retrospective	384	36	0.094
Asfaw and Afero (108)	2014	2014	Northeast	Tigray	Cattle	CS	440	141	0.32
Guduro and Desta (109)	2019	2016–2017	Southern	SNNPR	Cattle	CS	400	208	0.52
Kebede et al. (110)	2008	2008	Northeast	Tigray	Cattle	CS	5,194	1,146	0.221
Zelalem et al. (111)	2012	2007–2008	Central	AA	Sheep, cattle	CS	1,611	284	0.176
Kumsa (112)	2019	2015–2016	Central	AA	Cattle	CS	1,209	254	0.21
Negash et al. (31)	2013	2010–2011	Southwestern	Oromia	Cattle	CS	384	190	0.495
Abera et al. (113)	2013	2009–2010	Southern	SNNPR	Cattle	CS	564	205	0.363
Temam et al. (114)	2016	2015–2016	Southwestern	Oromia	Cattle	CS	400	218	0.545
Tibebu et al. (115)	2016	2010–2011	Northwestern	Amhara	Cattle	CS	415	149	0.359
Tkubet et al. (70)	2016	2014–2015	Northeast	Tigray	Cattle	CS	310	80	0.258
Abera and Teklebran (116)	2017	2016	Southern	SNNPR	Cattle	CS	446	50	0.112
Hailu et al. (117)	2016	2014–2015	Southwestern	Oromia	Cattle	CS	384	195	0.507
Nasr and Pal (118)	2016	2015	Northwestern	Amhara	Cattle	CS	400	134	0.335
Beyene and Hiko (119)	2019	2017–2018	Southwestern	Oromia	Cattle	CS	384	128	0.333
Atsede and Abebe (120)	2016	2015–2016	Southwestern	Oromia	Cattle	CS	384	118	0.307
Tadesse et al. (121)	2016	2013–2014	Northwestern	Amhara	Cattle	CS	384	72	0.187
Haftu and Kebede (122)	2016	2014–2015	Southwestern	Oromia	Cattle	CS	246	29	0.118
Belisty et al. (123)	2017	2015–2016	Northwest	Amara	Cattle	CS	620	134	0.216
Gemeda et al. (124)	2020	2018–2019	Southwestern	AA	Sheep, goat	CS	995	216	0.217
Yohannes (125)	2019	2017–2018	Southwestern	Oromia	Cattle	CS	395	159	0.402
Moje and Degefa (126)	2014	2011–2012	Southwestern	Oromia	Cattle	CS	405	41	0.101
Berhe (127)	2009	2006–2007	Northeast	Tigray	Cattle	CS	4,418	1,439	0.326
Dana et al. (128)	2021	2019–2020	Southwestern	Oromia	Cattle	CS	389	206	0.53
Akeberegn et al. (129)	2017	2017	Northern west	Amara	Cattle	CS	384	25	0.065
Andualem et al. (130)	2016	2014–2015	Central	AA	Cattle	CS	486	98	0.202
Serda and Jago (131)	2017	2014–2015	Southeastern	Oromia	Cattle	CS	450	243	0.54
Kebede et al. (132)	2009	2005–2006	Northwest	Amhara	Cattle, sheep	CS	760	176	0.2316
Kebede et al. (133)	2011	2007–2008	Northwest	Amhara	Cattle	CS	521	79	0.152
Mathewos et al. (134)	2022	2020–2021	Southeastern	SNNPR	Cattle	CS	384	69	0.18
Kebede et al. (135)	2009	2007–2008	Northwest	Amhara	Cattle	CS	413	202	0.489
Kebede et al. (110)	2009	2007–2008	Southeastern	SNNPR	Cattle	CS	400	64	0.16
Bekele and Butako (136)	2010	2009–2010	Southeastern	SNNPR	Cattle	CS	546	92	0.168

CS, cross-sectional study; SNNPR, South Nation and Nationalities of People; AA, Addis Ababa.

### Toxoplasmosis

In the present study, 14 articles involving a total of 6,957 animals were examined, with 2,889 of those animals diagnosed as positive for toxoplasmosis. According to Teshale et al. (32), the central region of Ethiopia exhibited the highest prevalence rate of 74.9% at the individual study level. In comparison, Esubalew et al. (33) reported a prevalence rate of 70.5% in the Amhara region, Northwest Ethiopia. With respect to the species of host animals, Esubalew et al. (33) reported a high prevalence of toxoplasmosis (70.5%) in the Amhara region in sheep, followed by goats (74.9%), as indicated by Teshale et al. (32), and cattle (59%), as reported by Maru et al. (50), in the Amhara region. Among the included studies, five did not state ethical considerations. The details of the articles are summarized in Table 3.

**Table 3:** Characteristics of the included studies (*n* = 14) toxoplasmosis.

First author	Publication year	Study year	G. Location	Region	Host	Ethical consideration	ST	Method diagnosis	Data collection method	Total examine	Positive	Prevalence
Tegegne et al. (137)	2016	2014–2015	Southwest	Oromia	Sheep, goat	Yes	Univariable-logistic regression	LAT	Serological assay	368	212	0.576
Tilahun et al. (138)	2018	2011–2013	Southwest	Oromia	Seep, goat, cattle	Not stated	Multivariable logistic regression	iELISA	Serological and questionnaire survey	778	260	0.334
Gebremedhin et al. (139)	2013	2010–2011	Southwest	Oromia	Sheep	Not stated	Multivariate logistic regression	ELISA	Serological and questionnaire survey	1,130	357	0.316
Maru et al. (50)	2016	2014–2015	Northwest	Amhara	Sheep, goat, cattle	Yes	Chi-square	LAT	Bioassay, serology and microscopy	335	187	0.558
Zewdu et al. (140)	2013	2010–2011	Northwest	Amhara	Goat	Yes	Multivariable logistic regression	ELISA	Serological and questionnaire survey	927	183	0.197
Teshale et al. (32)	2007	2005–2006	Central	AA	Goat	Not stated	Multistage and cluster sampling	DAT	Serological assay	641	480	0.749
Negash et al. (141)	2004	1999–2000	Northeast	Tigray	Sheep, goat	Yes	Simple random	DAT	Serological assay	174	75	0.431
Gebremedhin and Gizaw (142)	2014	2013–2014	Southern	SNNPR	Sheep, goat	Yes	Mantel-haenszell Chi-square	iELISA	Serological assay	184	48	0.261
Gebremedhin et al. (143)	2014	2011–2012	Northeast	Tigray	Sheep, goat	Yes	Multivariable logistic regression	DAT	Serological questionnaire survey	628	111	0.177
Jilo et al. (144)	2021	2016–2017	Southern	Oromia	Sheep, goat	Yes	Simple random	LAT	Serological and questionnaire survey	400	211	0.5275
Getachew et al. (61)	2016	2011–2012	Central	AA	Sheep, goat, cattle	Not stated	Simple random	DAT	Serological and questionnaire survey	278	97	0.349
Esubalew et al. (33)	2022	2018–2019	Northwest	Amhara	Sheep, goat	Yes	Cluster random sampling	LAT	Serological and questionnaire survey	576	406	0.705
Gashaw et al. (51)	2023	2019–2021	Northwest	Amhara	Goat	Yes	Systematic random sampling	LAT	Serological and questionnaire survey	150	65	0.433
Zewde et al. (145)	2022	2020–2022	Southwest	Oromia	Sheep, goat	Not stated	Simple random	ELISA	Serological assay	388	197	0.508

LAT, latex agglutination test; ST, Sampling technique; IELISA, Indirect enzyme-linked immunoassay; DAT, direct agglutination test; SNNP, South Nation and Nationalities of peoples; G. Location, geographical location.

### Cryptosporidiosis

A total of 19 studies were discovered for the estimation of the pooled prevalence of cryptosporidiosis. The majority of the studies are located in the southern region of Ethiopia, specifically within the Oromia region. The study subjects included calves, kids, lambs, and adult sheep and cattle, which consisted of a total of 6,972 and 959 cryptosporidiosis infections (event). Notably, most of the literature has focused on calves and lambs. The most frequently isolated species were *C. bovis*, *C. andersoni*, *C. ubiquitum*, and *ryane*. The southwest region of Ethiopia, specifically the Oromia regional state, recorded a high prevalence rate (24%) of cryptosporidiosis with calves documented by Gashaw et al. (51) (Table 4).

**Table 4:** Characteristics (*n* = 19) of the included studies of cryptosporidiosis.

First author	Study year	Location	Region	Host	Total examine	Positive	Prevalence	Method detection	Molecular characterization	ST	Data collection	Ethical consideration
Hailu et al. (20)	2017–2018	Southern	SNNPR	Calves	330	43	0.13	DIFAT	Not done	SR	Mixed	Yes
Wegayehu et al. (18)	2009	Southwest	Oromia	Calves	384	30	0.0781	MZN	Not done	MRS	Quantitative	Yes
Kifleyohannes et al. (52)	2018–2019	Northeast	Tigray	Kids, calves, lambs	726	53	0.073	MZN, PCR	*C. ubiquitum, C. bovis, C. andersoni, C. ryanae*	COS	Mixed	Yes
Manyazewal et al. (53)	2018	Central	AA	Cattle	392	73	0.1862	MZN, PCR, PCR-RFLP	*C. parvum, C. andersoni*	SR	Quantitative	Yes
Abebe et al. (55)	2004–2005	Central	AA	Calves	580	102	0.176	SF, MZN	Not done	CLS	Mixed	Not stated
Ayele et al. (146)	2014–2015	Northwest	Amhara	Calves	360	67	0.186	SF, MZN	Not done	SR	Quantitative	Yes
Birhanu et al. (147)	2014–2015	Southwest	Oromia	Calves	384	92	0.24	MZN	Not done	SR	Mixed	Not stated
Manyazewal et al. (148)	2014–2015	Southwest	Oromia	Calves	270	40	0.148	MZN, PCR	*C. bovis, C. ryane, C. andersoni*	PS	Quantitative	Yes
Wegayehu et al. (149)	2014	Southwest	Oromia	Calves	449	91	0.203	MZN, PCR	*C. parvum, C. andersoni*	SR	Mixed	Yes
Gashaw et al. (150)	2018–2019	Southwest	Oromia	Cattle	378	41	0.108	MZN	Not done	SR	Mixed	Yes
Wegayehu et al. (149)	2014	Southwest	Oromia	Lambs	389	8	0.021	MZN, nested PCR	*C. ubiquitum*	SR	Quantitative	Yes
Berhanu et al. (151)	2021–2022	Southwest	Oromia	Cattle	434	76	0.175	SF, MZN	Not done	SR	Mixed	Yes
Ali et al. (152)	2017–2018	Southern	SNNPR	Sheep	384	31	0.081	SF, MZN	Not done	SR	Mixed	Not stated
Ebiyo and Haile (153)	2020–2021	Southwest	Oromia	Calves	384	53	0.138	MZN	Not done	SYR	Quantitative	Yes
Regassa et al. (13)	2010–2011	Southwest	Oromia	Calves, lambs, kids	237	56	0.236	SF, MZN	Not done	SYR	Quantitative	Yes
Ayana and Alemu (154)	2014–2015	Central	AA	Calves, lambs, kids	364	27	0.136	MZN	Not done	SYR	Mixed	Yes
Wudu et al. (155)	2004	Southwest	Oromia	Calves	55	4	0.073	MZN	Not done	SR	Quantitative	Not stated
Tekle (156)	2020–2021	Northwest	Amhara	Calves	193	30	0.155	MZN	Not done	SR	Mixed	Not stated
Belfa et al. (157)	2021–2022	Central	Oromia	Calves	279	42	0.151	MZN	Not done	CLS	Mixed	Not stated

ST, sampling technique; MZN, modified Zeehl–Nielsen; CLS, cluster sampling; COS, convenient sampling; SR, simple random sampling; SYR, systematic random sampling; MRS, multistage sampling; PS, purposive sampling; mixed, quantitative and qualitative; SF, flotation; DIFAT, direct immuno-flurecent antigen test.

### Diagnostic methods used across studies

The diagnostic approaches varied across the four parasitic diseases, with post-mortem meat inspection, serological tests, microscopy, and molecular techniques being the primary methods. Post-mortem meat inspection was the gold standard for diagnosing bovine cysticercosis and cystic echinococcosis, as both diseases were primarily identified through abattoir-based surveys (Tables 1, 2). The presence of cystic lesions in infected organs served as a key diagnostic indicator. Since data were collected from slaughtered animals, ethical approval was generally not required for these studies. For toxoplasmosis, serological tests were the primary diagnostic tool, with ELISA, Latex Agglutination Test (LAT), and Direct Agglutination Test (DAT) commonly used across studies (Table 3). These methods detect specific antibodies in blood samples, making them suitable for screening live animals. In several studies, serological tests were supplemented with questionnaire surveys to assess risk factors, transmission dynamics, and disease prevalence in different livestock populations.

Cryptosporidiosis was predominantly diagnosed using microscopy (modified Zeehl–Nielsen), which detects oocysts in fecal samples. Some studies employed PCR and nested PCR to achieve molecular characterization of *Cryptosporidium* species, including *C. bovis, C. andersoni, C. ubiquitum*, and *C. ryanae* (18, 52, 53) as shown Table 4. However, molecular diagnostics were used in only a limited number of studies. Additionally, questionnaire surveys were commonly employed as a supplementary diagnostic tool across all four parasitic diseases. These surveys provided valuable insights into risk factors, transmission patterns, and disease awareness among livestock owners. By integrating observational, serological, molecular, and questionnaire-based approaches, the studies provided a comprehensive assessment of zoonotic parasitic diseases in Ethiopia.

### Summary of findings from the meta-analysis

This meta-analysis of the included studies provides an in-depth assessment of the pooled prevalence, heterogeneity, and potential sources of variability for each parasitic disease. The findings highlight significant variations in prevalence rates across regions, host species, and diagnostic methods, emphasizing the need for subgroup analyses to better understand disease distribution and impact. The substantial heterogeneity were observed in this meta-analysis is likely driven by several factors, including variations in study design, host species, diagnostic techniques, and geographic distribution. The heterogeneity test confirmed significant differences between species, suggesting that biological and ecological factors play a major role in prevalence estimates.

Regional differences in prevalence are likely influenced by climate, altitude, and management practices. Warmer, humid environments may enhance oocyst survival and transmission, while variations in animal husbandry (e.g., free grazing vs. confined feeding) impact exposure risk. Differences in veterinary healthcare infrastructure further affect disease detection and control.

Heterogeneity also arises from methodological variations among studies. Differences in sampling strategies (random, convenience, stratified), population characteristics, and study periods contribute to variability. Sample sizes ranged from small farms to large epidemiological studies, affecting prevalence estimates. Study designs varied, with some focusing on controlled farm settings and others on free-grazing animals, leading to inconsistencies in exposure risk. Additionally, the use of different diagnostic assays influenced reported prevalence rates.

The following sections summarize the key results for each disease, incorporating heterogeneity measures, statistical findings, and publication bias assessments.

### Meta-analysis results for toxoplasmosis

**Figure 2:**
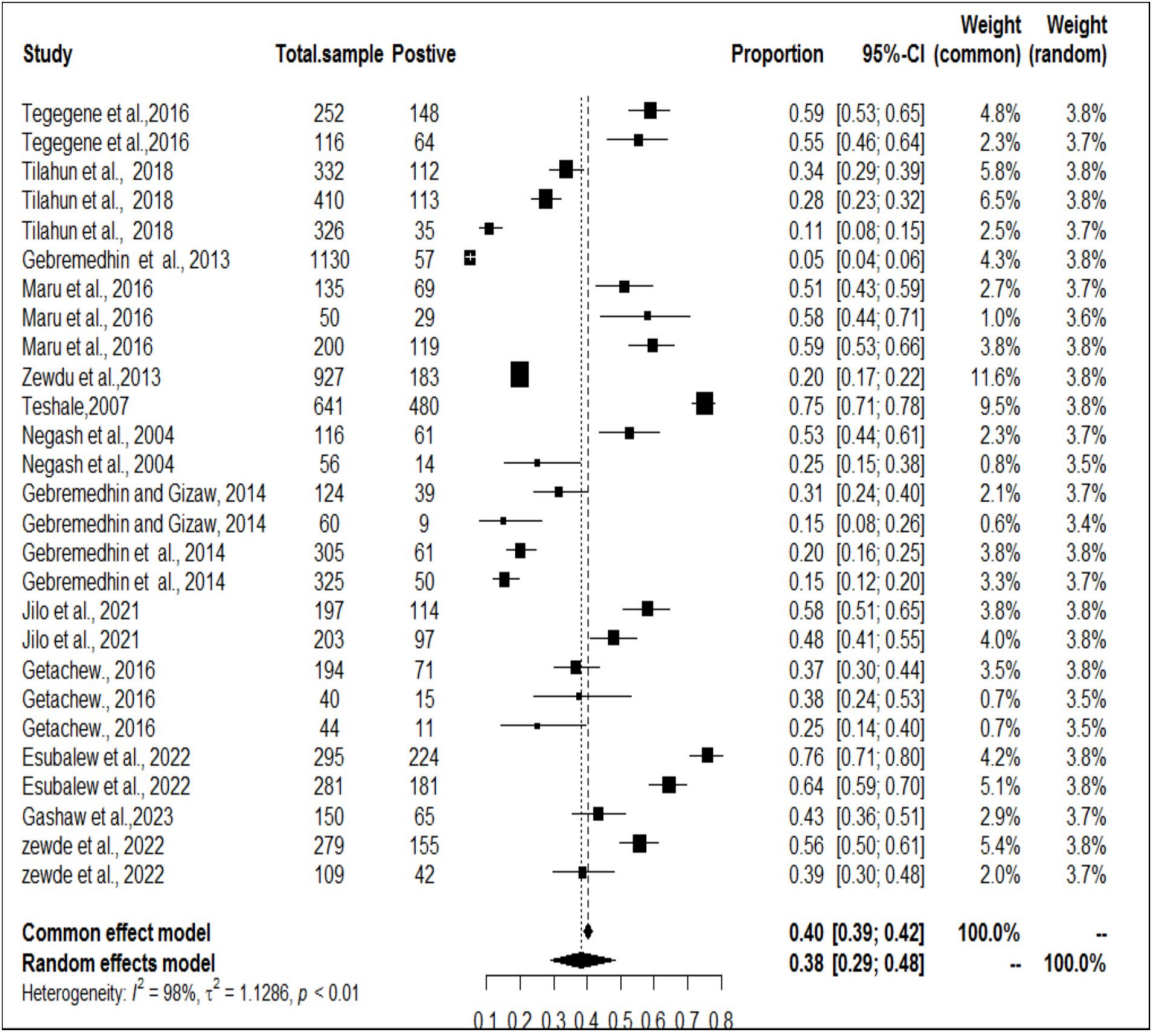
Random forest plots with overall prevalence for toxoplasmosis in ruminants.

**Figure 3:**
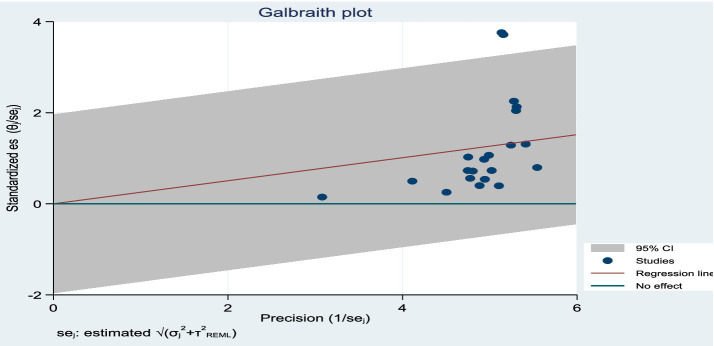
Galbraith plot showing the heterogeneity of the prevalence of toxoplasmosis.

**Figure 4:**
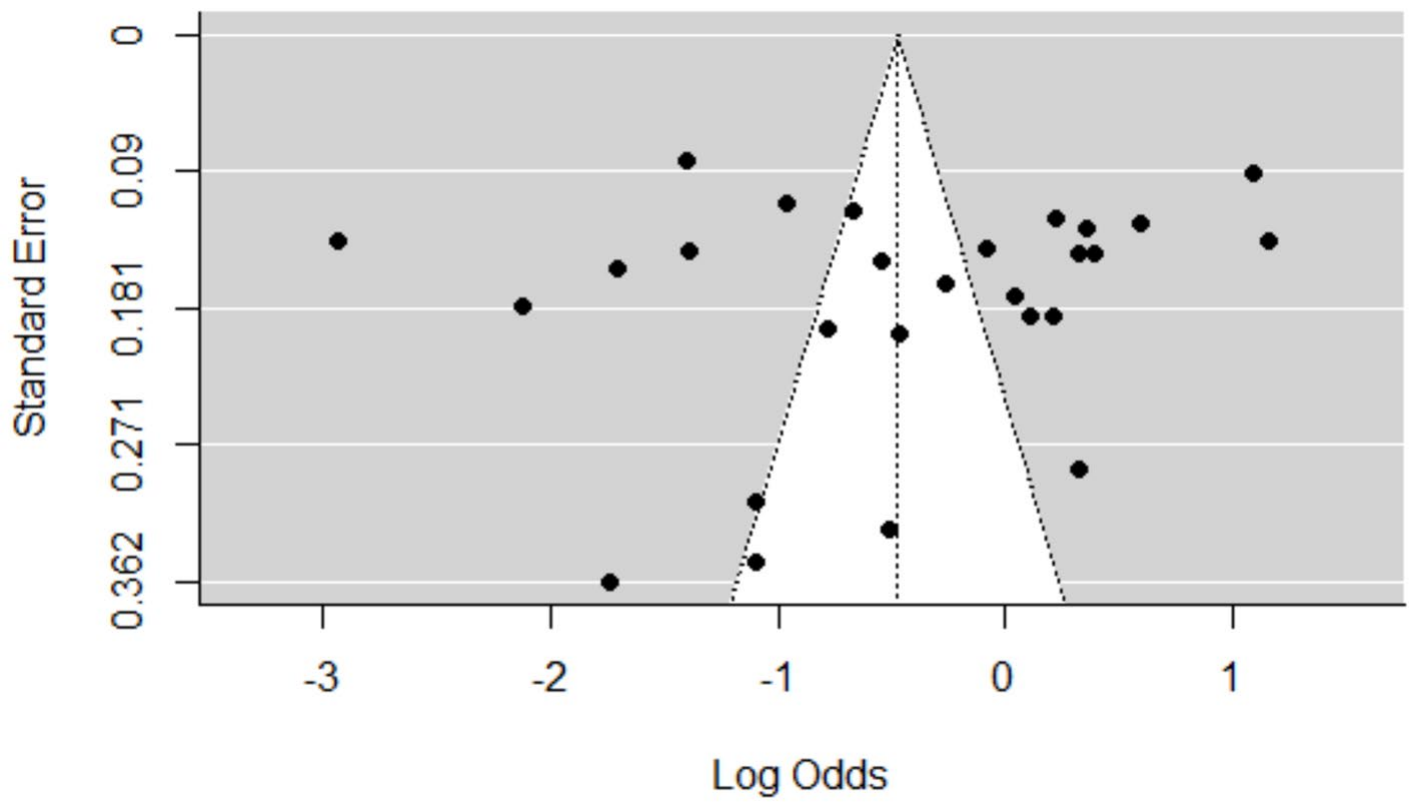
Funnel plots of the standard error by log odds of the prevalence of toxoplasmosis.

The estimated pooled prevalence was 38% (95% CI: 29–48%, Figure 2), indicating a relatively high burden of toxoplasmosis in domestic ruminants. However, significant heterogeneity was observed (*I*^2^ = 98%; τ^2^ = 1.1286; *p* < 0.01), suggesting variations in study design, host species, or diagnostic methods across the included studies. The Galbraith plot confirmed high inter-study heterogeneity, as nearly 95% of the studies fell outside the confidence interval (Figure 3). Further subgroup analyses based on host species and geographic location are essential to understand which animal groups or regions experience higher prevalence rates. Despite the heterogeneity, no significant publication bias was detected, as indicated by Egger’s regression asymmetry coefficient (*b* = 2.38; 95% CI: −2.19, 4.43; *p* > 0.05). The funnel plots (Figure 4) also demonstrated a symmetrical distribution, reinforcing the robustness of the meta-analysis findings.

### Sensitivity

**Figure 5:**
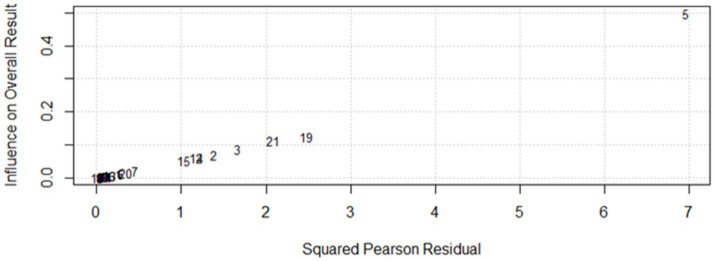
Diagnosis of potential outliers from the included studies.

Baujat diagnostic plots (54) was used to detect studies that overly contribute to the heterogeneity in a meta-analysis. The plot shows the contribution of each study to the overall heterogeneity (as measured by Cochran’s Q) on the horizontal axis and its influence on the pooled effect size on the vertical axis. Accordingly, a study by author ID number 5 which denotes Sheferaw and Abdu (1) was found to be an influential study graphically which contributed overly to the overall heterogeneity, though it did not affect the pooled effect size significantly as shown in the leave-one-out meta-analysis result (Figure 5). High heterogeneity (*I*^2^ = 98%) suggests further subgroup is needed to explore sources of variability. Finally, a leave-one-out analysis was conducted to evaluate the influence of a single study on the overall effect size estimate. In this analysis, a specific study was excluded, and a meta-analysis was performed on the remaining (*n*−1) studies. If the confidence interval of the study did not encompass the overall effect size estimate, it was deemed that the study had a substantial impact on it. In this study, the overall effect size estimate was 0.38, and it fell within the confidence intervals of all the studies. As a result, excluding one study did not have a substantial impact on the overall effect size estimate (Figure 2). Similarly, sensitivity analyses have been conducted in a comparable manner for toxoploamosis disease studies to assess the impact of individual studies on the overall pooled prevalence estimates. For example, in the study by Abebe et al. (55), the graphical sensitivity analysis indicated that this study had a notable influence on the reported prevalence of cryptosporidiosis, suggesting that its inclusion may have contributed to variations in prevalence estimates. Likewise, in the study by Higgins and Thompson (47), sensitivity analysis revealed that this study was influential in the context of cystic echinococcosis, demonstrating a similar pattern of influence.

However, despite these individual studies showing some degree of influence in graphical representations, their exclusion did not significantly alter the overall pooled prevalence estimates. This suggests that while certain studies may introduce variability at a localized level, the robustness of the meta-analysis remains intact, and the general conclusions drawn from the pooled data are not substantially affected. These findings reinforce the stability of the prevalence estimates and highlight the reliability of our analytical approach in accounting for potential outliers.

### Meta-analysis results for cryptosporidiosis

**Figure 6:**
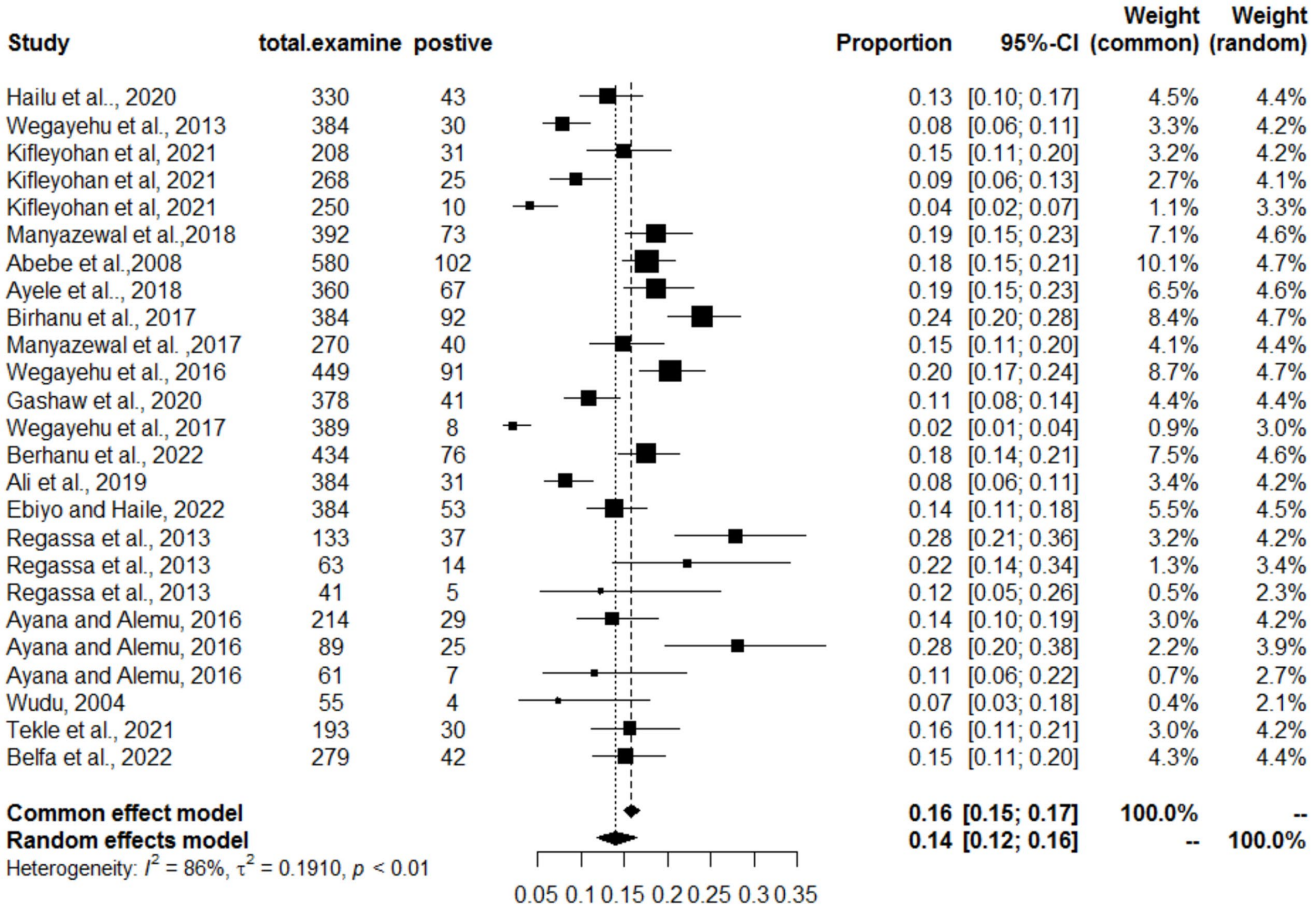
Random forest plots with overall prevalence of cryptosporidiosis in animals.

**Figure 7:**
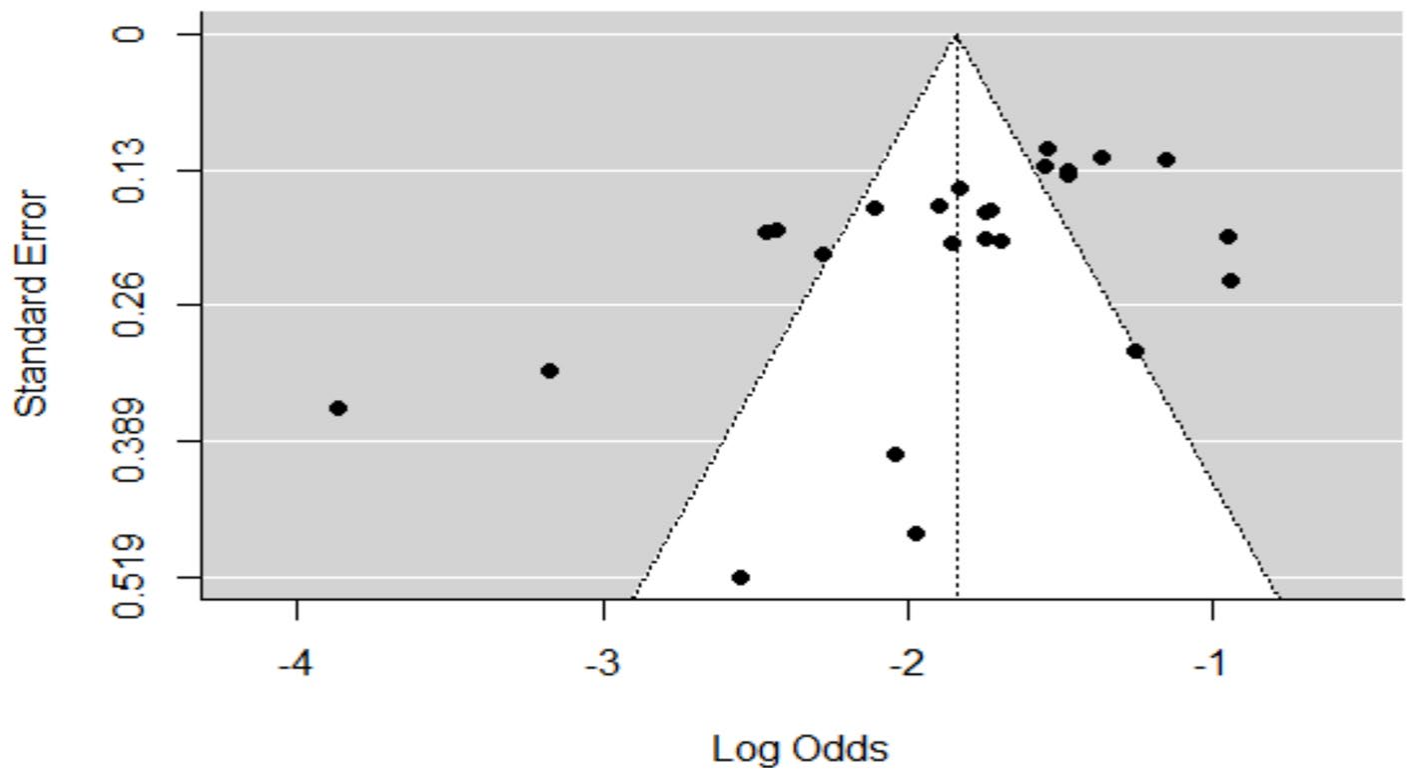
Funnel plots of the standard error by log odds of the prevalence of cryptosporidiosis.

For cryptosporidiosis, 19 studies were analyzed, revealing a pooled prevalence of 14% (95% CI: 12–16%). The heterogeneity was substantial (*I*^2^ = 86%; τ^2^ = 0.1910; *p* < 0.01), indicating that study-level differences significantly influenced the estimated prevalence rates. The forest plot (Figure 6) illustrates variations in prevalence across studies, while the funnel plot (Figure 7) suggests that the studies were evenly distributed without asymmetry, indicating no significant publication bias. The differences in diagnostic techniques (microscopy vs. molecular methods) and host species (calves, lambs, and adult ruminants) may contribute to the heterogeneity. Additionally, environmental factors such as hygiene, management practices, and seasonal variations could also influence prevalence rates. The Egger regression test (*b* = 1.2953; CI: 0.1792, 1.4114; *p* = 0.2007) further confirmed the absence of statistically significant publication bias in the meta-analysis.

### Meta-analysis results for bovine cysticercosis

**Figure 8:**
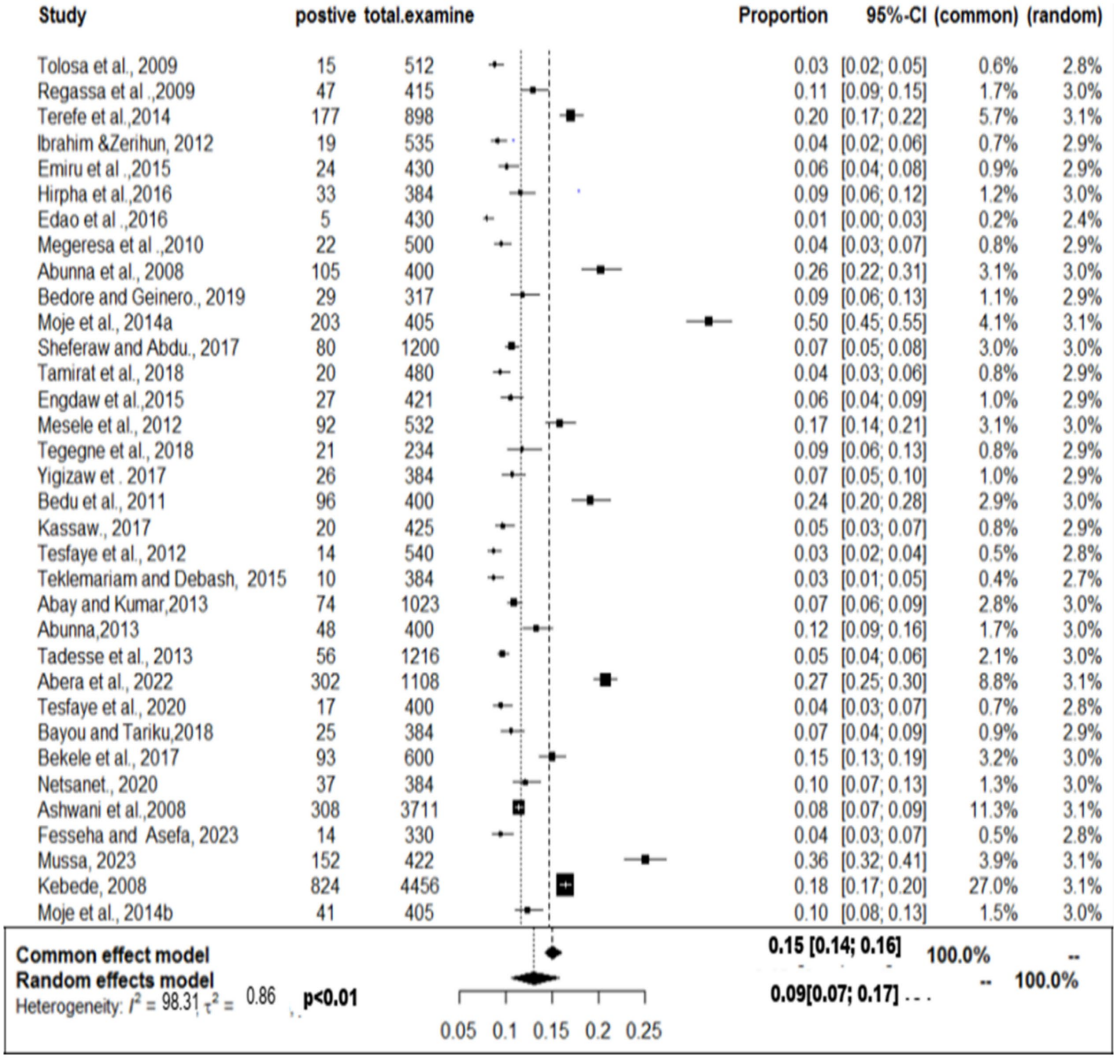
Random forest plots showing the overall prevalence of bovine cysticercosis in animals.

**Figure 9:**
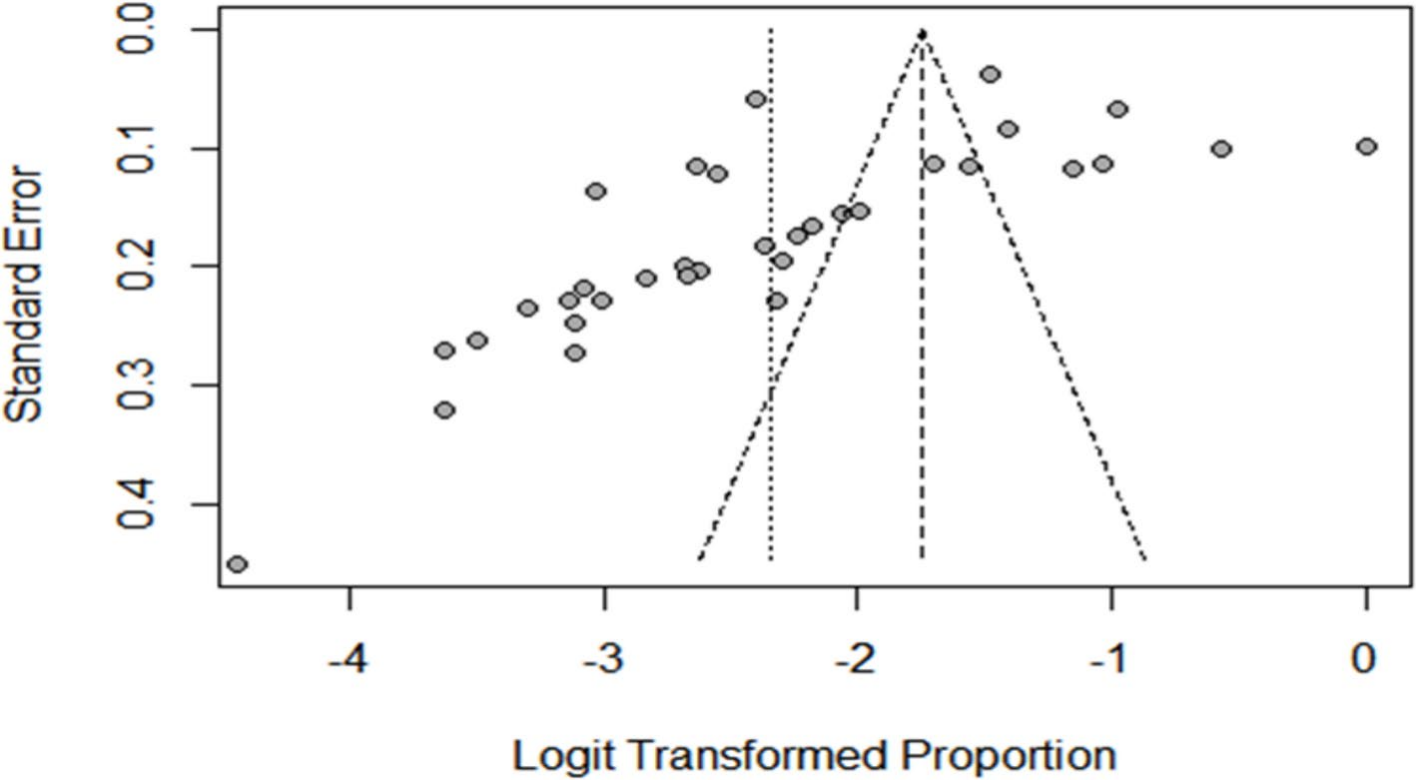
Funnel plot of standard errors by logit of the prevalence of bovine cysticercosis.

The overall pooled prevalence was 9% (95% CI: 7–17%: Figure 8), highlighting a moderate burden of bovine cysticercosis in Ethiopia. The analysis showed high between-study variability (τ^2^ = 0.861; *H*^2^ = 59; *I*^2^ = 98.31%), reinforcing the need for subgroup analyses based on host characteristics and geographic factors. The funnel plot (Figure 9) suggested asymmetry, indicating the possibility of publication bias, which was confirmed by Egger’s regression test (*b* = 0.3229; 95% CI: 0.1792, 0.4114; *p* = 0.02812). This suggests that smaller studies may be underreported or that there is selective reporting of significant results, potentially influencing the pooled prevalence estimate.

### Meta-analysis results for cystic echinococcosis

**Figure 10:**
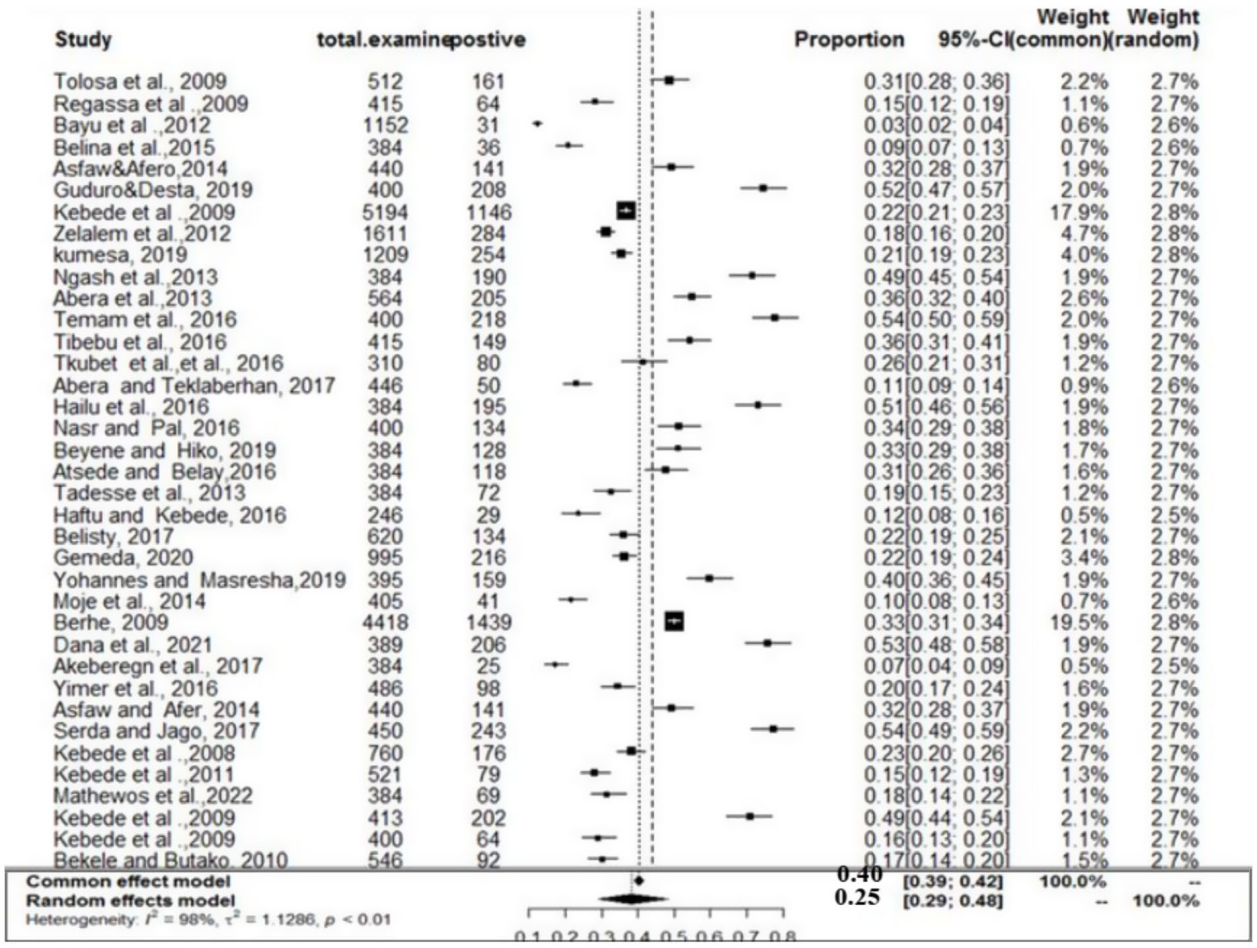
Random forest plots showing the overall prevalence of cystic echinocococcosis in domestic food ruminants.

**Figure 11:**
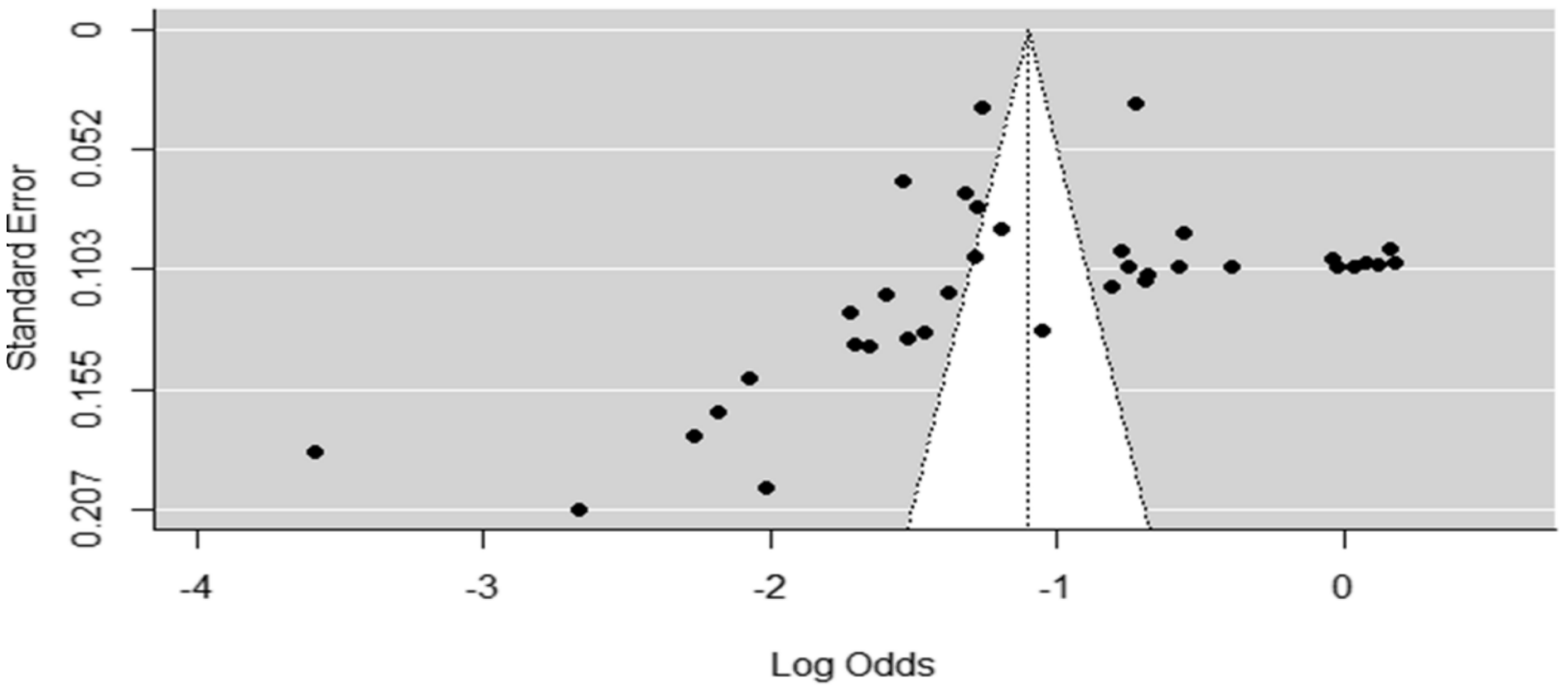
Funnel plot of standard error by log odds of prevalence estimates of cystic echinococcosis.

The pooled prevalence was estimated at 25% (95% CI: 29–48%: Figure 10), indicating a substantial disease burden among domestic ruminants. High heterogeneity (τ^2^ = 0.7104; *I*^2^ = 98.0%) was observed, likely due to differences in study locations, sample sizes, and diagnostic approaches. The funnel plot (Figure 11) showed no asymmetry, suggesting that smaller studies were not systematically underrepresented. Similarly, Egger’s test (*b* = −2.58; 95% CI: 0.17–0.31; *p* = 0.08075) confirmed that publication bias was not statistically significant. The high prevalence and widespread distribution of cystic echinococcosis underscore its public health and economic impact, reinforcing the need for improved disease control strategies in Ethiopia.

### Subgroup analysis

A subgroup analysis of each of the four parasitic diseases (toxoplasmosis, cryptosporidiosis, bovine cysticercosis and cystic echinococosis) was conducted on the basis of year, species, and region. All of the analyses of exploratory outcomes showed considerable heterogeneity (*I*^2^ > 71). Significant statistical heterogeneity in the subgroup analysis reveals a likely interaction among exploratory variables.

### Subanalysis results of toxoplasmosis

**Figure 12:**
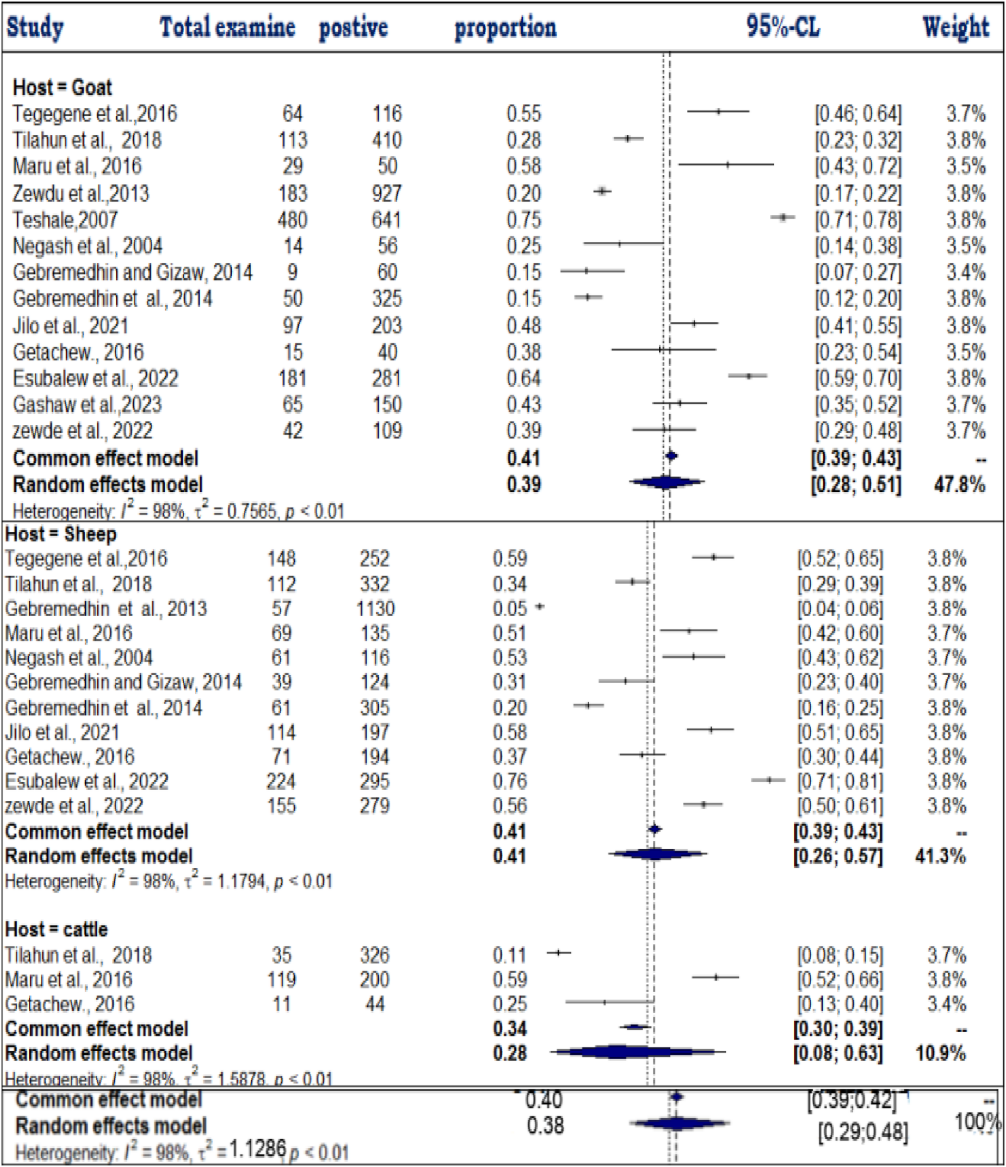
Prevalence of toxoplasmosis among different species of animals.

The subanalysis of toxoplasmosis prevalence across different animal species revealed substantial variability, which we now interpret more clearly. The studies were categorized into three groups based on animal species: goats (*n* = 13), sheep (*n* = 11), and cattle (*n* = 3). The pooled prevalence estimates for toxoplasmosis varied across these species, with sheep showing the highest prevalence at 41% (95% CI: 26–57%; *I*^2^ = 98%), followed by goats at 39% (95% CI: 28–51%) and cattle at 28% (95% CI: 8–63%) (Figure 12). Despite the high level of within-study variability across all species (*I*^2^ = 98%), the heterogeneity test revealed statistically significant differences (*Q* = 180.28; D.F. = 18; *p* < 0.001). However, no significant difference in the number of studies between species was observed (*Q* = 0.8; D.F. = 2; *p* = 0.7855), indicating that the observed variation in prevalence is not due to the number of studies per group but likely reflects other factors, such as ecological or methodological influences.

**Figure 13:**
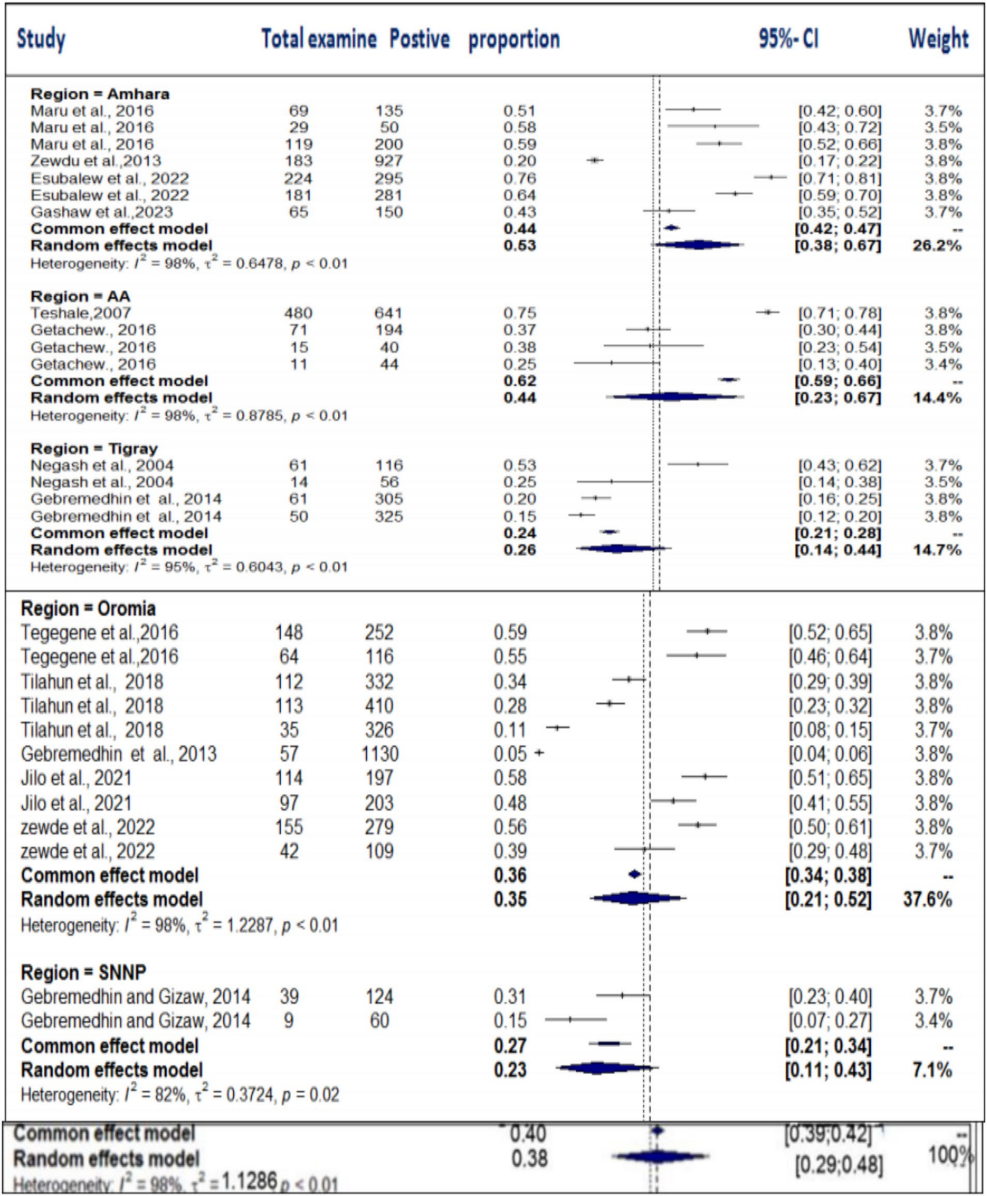
Subgroup analysis of the distribution of toxoplasmosis based on the study region.

The regional variability analysis also provided important insights. We found similar levels of variability (*I*^2^ = 98%) across the Amhara, AA, and Oromia regions. The pooled prevalence for toxoplasmosis was highest in the Amhara region (53%), followed by AA (44%), Tigray (26%), Oromia (35%), and SNNP (23%) (Figure 13). The regional heterogeneity test (*Q* = 8.21; D.F. = 4; *p* < 0.00841) indicated statistically significant differences, suggesting that regional factors, such as climate and management practices, may influence the prevalence of toxoplasmosis.

**Figure 14:**
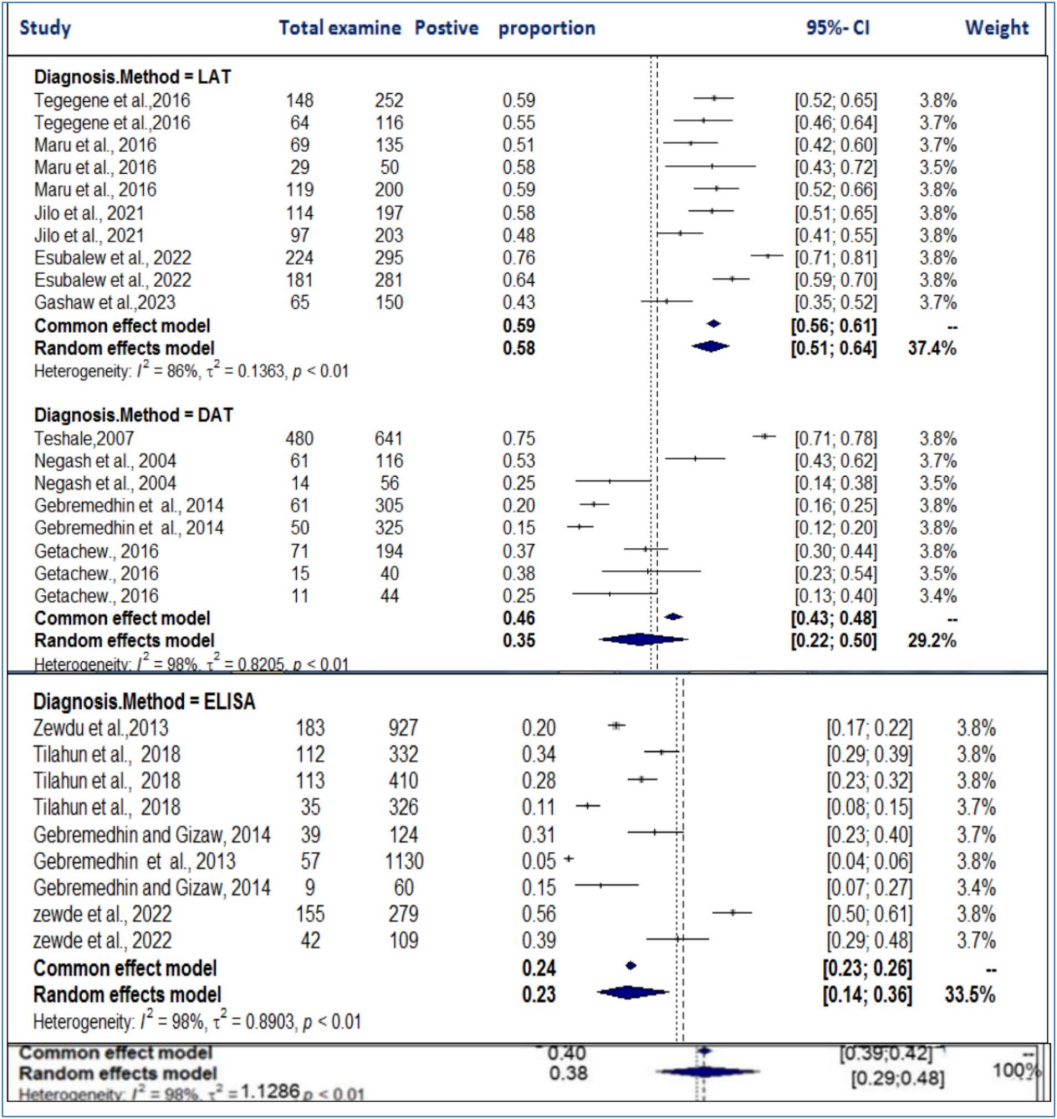
Subanalysis of the pooled prevalence of toxoplasmosis by method of diagnosis.

Furthermore, subgroup analysis based on diagnostic techniques (LAT, DAT, and ELISA) revealed considerable heterogeneity (*I*^2^ > 86%) across all methods (Figure 14). The highest degree of heterogeneity (*I*^2^ = 98%) was observed in studies using the DAT and ELISA diagnostic methods. The pooled prevalence was highest in studies using the LAT method (58%), followed by DAT (35%) and ELISA (23%). The subgroup difference test showed a statistically significant group effect (*Q* = 23.49; D.F. = 2; *p* < 0.0001), suggesting that the choice of diagnostic method plays a significant role in the variation in prevalence estimates. We have emphasized the potential clinical and ecological implications of these findings and noted that the variability observed may reflect underlying biological, environmental, or methodological factors that were not fully addressed in the analysis. Regional factors such as climate, management practices, and differences in diagnostic methods could contribute to the observed discrepancies in prevalence estimates. We also acknowledge that further research is needed to explore the biological, ecological, and methodological factors that contribute to these variations. This additional research would help provide a more comprehensive understanding of the true prevalence of toxoplasmosis and its implications for livestock health and management.

### Subanalysis results of cryptosporidiosis

**Figure 15:**
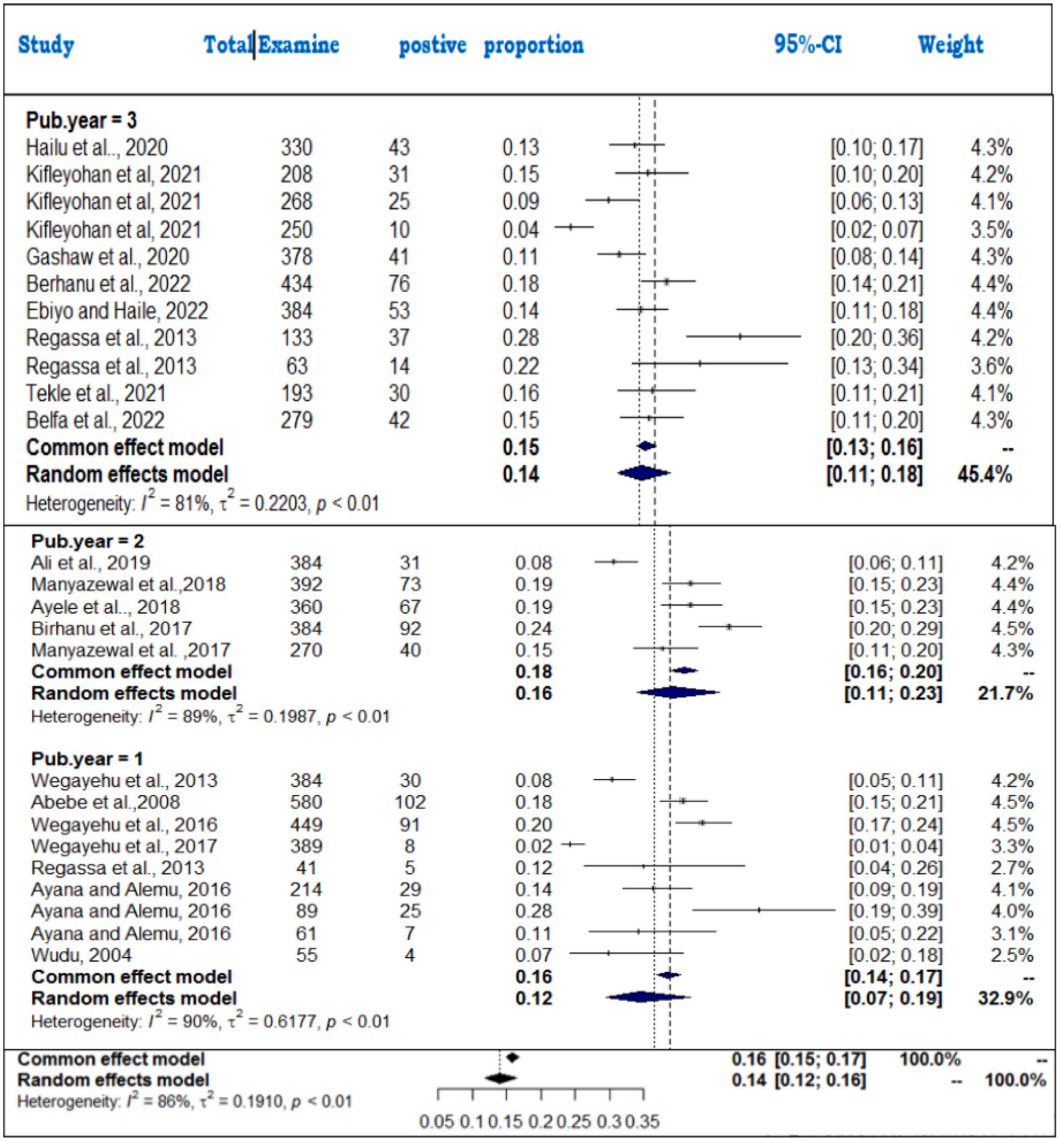
Forest plots of subgroup analysis of cryptosporidiosis by publication year. 1 = 2004–2016, 2 = 2017–2019, 3 = 2020–2023.

The subanalysis of cryptosporidiosis prevalence across different publication years revealed the highest prevalence between 2017 and 2019 (16%) compared to other periods (14% for 2020–2023 and 12% for 2004–2016) (Figure 15). Statistically significant differences were found between the time periods (*Q* = 8.69; D.F. = 2; *p* = 0.001), with high heterogeneity (*I*^2^ = 89% for 2017–2019, *I*^2^ = 81% for 2020–2023, and *I*^2^ = 89% for 2004–2016). The variation in prevalence could reflect improvements in detection methods over time, but further investigation is needed to understand the driving factors.

**Figure 16:**
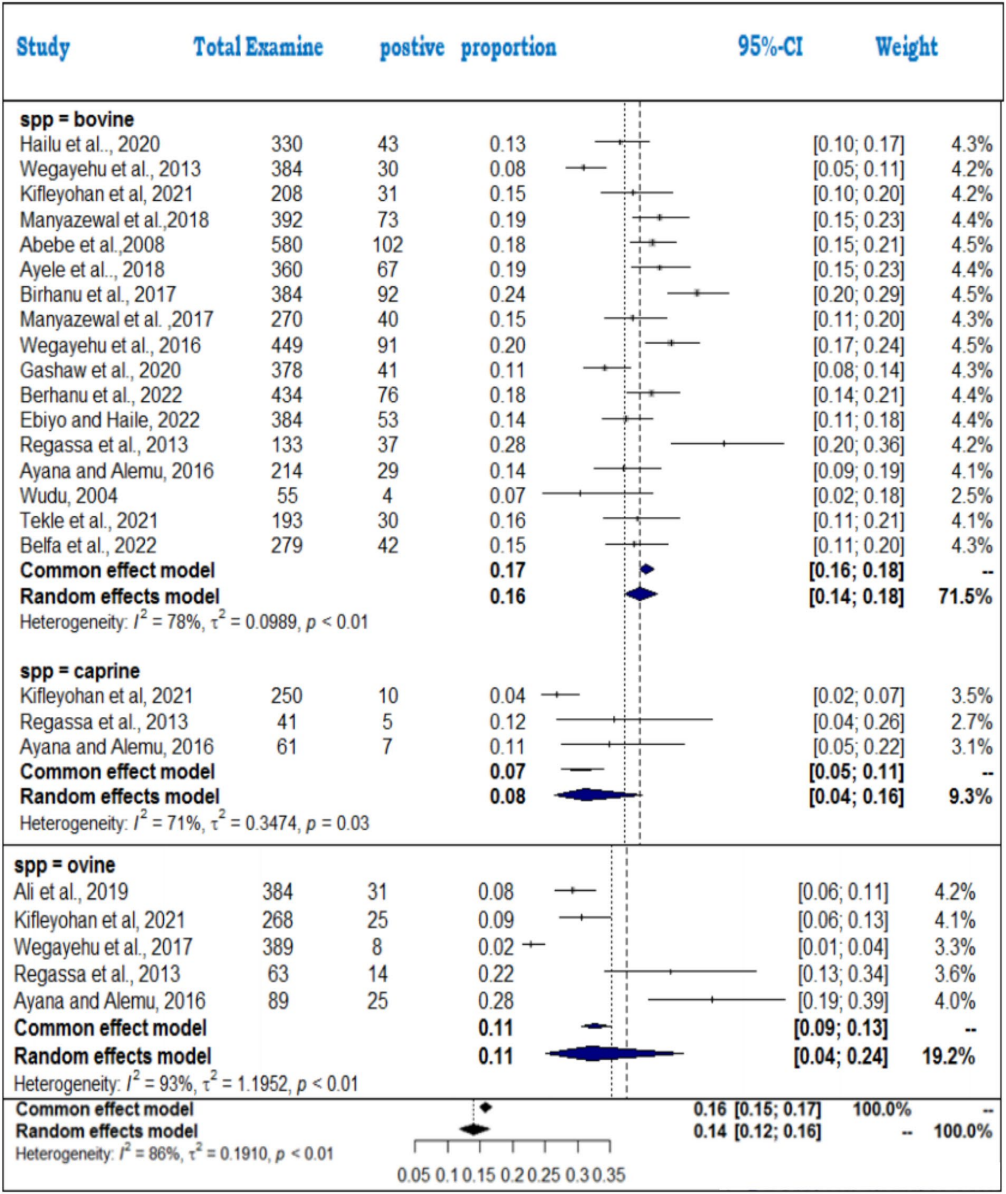
Subgroup analysis of cryptosporidiosis by animal species.

For the animal species analysis, bovines had the highest pooled prevalence (16%), followed by ovines (11%) and caprines (8%) (Figure 16). The heterogeneity was highest in ovine (*I*^2^ = 93%), followed by bovines (*I*^2^ = 78%) and caprines (*I*^2^ = 71%). The significant subgroup effect (*Q* = 33.3; D.F. = 2; *p* < 0.001) indicates that species differences play a key role in prevalence rates, and specific factors, such as immune responses and management practices, likely contribute to this variability.

**Figure 17:**
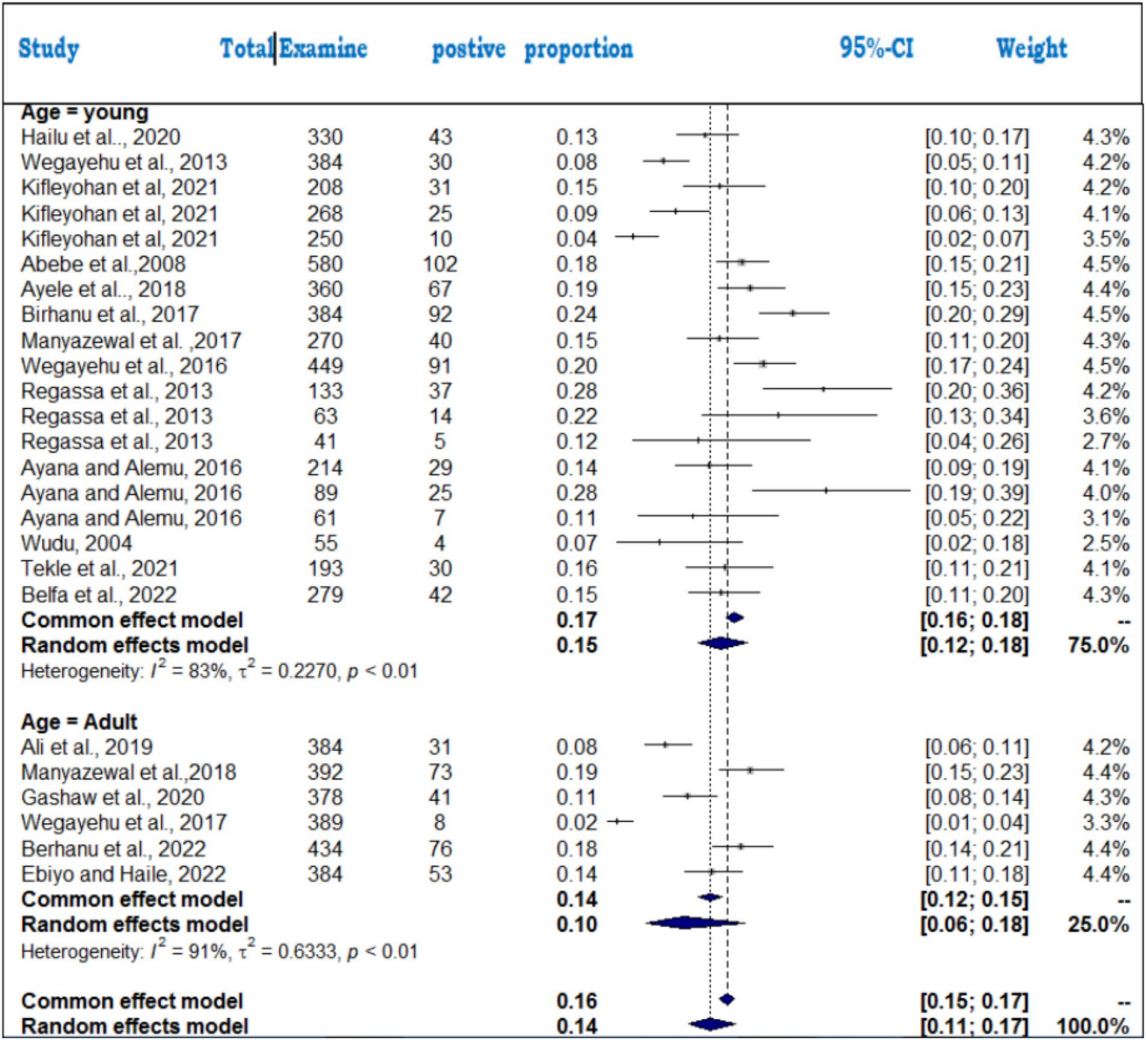
Subgroup analysis by age category to examine cryptosporidiosis in foods of animal origin.

The age category subanalysis showed more variability in adults (*I*^2^ = 91%) than in young animals (*I*^2^ = 83%). The pooled prevalence for young animals (15%) was slightly higher than for adults (14%) (Figure 17), suggesting that age-related differences might influence susceptibility to cryptosporidiosis. These findings emphasize the need for further exploration of factors such as species, age, and time period to better understand cryptosporidiosis prevalence and improve control strategies.

### Meta-regression

Uni-variable meta-regression was conducted with animal species (goat, sheep and cattle), and region, sample size (continuous variable) and diagnosis method used as categorical variables using the mixed effect model, in case of toxoplasmosis (Table 3). These variables were subjected to assessment to see a linear relationship with the dependent variable that is the effect sizes. Those variables with a *p*-values < 0.1 were used in the multivariable meta-regression analysis. Only region and diagnostic methods were found to be significant at *p*-values < 0.1 and therefore were included in the final multivariable analysis. The multivariable meta-regression model as a whole explained 21.96% amount of heterogeneity, while using diagnostic method and regional variability. Even when controlling for diagnostic methods compared to antibody detections (Table 5).

**Table 5:** Final multivariable meta-regression model for toxoplasmosis.

Variables	Coefficient (95% CI)	*P*-value	Adjusted *R*^2^
Diagnostic methods			21.96%
DAT	Ref.		
LAT	6.8845 (2.0784, 11.6906)	0.0102	
ELISA	7.5426 (2.3562, 12.7290)	0.0094	
Region			34.7%
Amhara	Ref.		
AA	−6.5243 (−11.3697, −1.6788)	0.0129	
Oromia	0.26 [−0.504, 1.03]	0.5029	
SNNP	0.85 [0.138, 1.55]		
Tigray	0.26 [−0.504, 1.03]	0.01303	
Sample size	−0.0008 (−0.0034, 0.0019)	0.5353	4.7%

*R*^2^, Coefficient of determination.

In case of cryptosporidiosis meta-regression was performed based on animal species (bovine, ovne and caprine), publication year (2004–2016, 2017–2019, and 2020–2023) and age category of the animals (Adult and Yung). Unfortunately, all the moderators were found to be significant at *p*-values < 0.1 at univariable meta-regression. Therefore, all of the three variables were included in multivariable meta-regression analysis (Table 6).

**Table 6:** Final multivariable meta-regression model (cryptosporidiosis).

Variables	Component	Coefficient	95%CI	*P*-value	Adjusted *R*^2^
Publication year	Intercept	−1.7494	2.2691–1.2297	<0.0001	0.2651
	2004–2016	Ref			23%
	2017–2019	0.5992	−0.2325, 1.4308	0.01579	
	2020–2023	1.6383	0.5072, 2.7693	0.0045	
Age	Adult	Ref.			18.4%
	Yung	−0.4068	−1.5234, 0.7098	0.04752	
Animal species	Bovine	Ref.			28.5%
	Caprine	−0.6475	3.0017, 1.7067	0.5898	
	Sheep	0.0880	−1.4906, 1.6665	0.9130	

### Subanalysis results of cysticercosis and cystic echinococcosis

Subgroup analysis of bovine cysticercosis and cystic echinococcosis based on study region and publication year was performed. The results of the subanalysis, such as the heterogeneity (I^2^), proportion, 95% CI, and test of between-study heterogeneity (Q), are summarized in Table 7. The subgroup difference test suggested that there was a statistically significant subgroup effect (*Q* = 11.69; DF = 1; *p* = 0.0029), implying that the publication year category was significantly associated with the prevalence of bovine cysticercosis in cattle, whereas the subgroup difference test suggested that there was a statistically significant subgroup effect (*Q* = 11.69; DF = 1; *p* = 0.0029) in the study region and that the prevalence of cystic echinococcosis (*Q* = 6.67; df = 4; *p* = 0.023).

**Table 7:** Summary of the results of the subgroup analysis of bovine cysticercosis and cystic echinococcosis according to study region and publication year.

Moderators		No studies	Proportion	95%CI	tau^2^	*I*^2^ (%)	Test for subgroup differences
Bovinecysticercosis							
Region wise	SNNPR	7	12%	(0.057; 0.234)	1.1723	97	*Q* = 5.94; df = 4 *p* = 0.2037
	Oromia	13	9%	(0.0510; 0.158)	1.2863	97	
	Tigray	2	8%	(0.0713; 0.090)	0.0021	3	
	AA	4	5%	(0.026; 0.077)	0.2728	84	
	Amhara	8	16%	(0.0579; 0.128)	0.3570	97	
Year wise	(≤ 2015)	19	8.12%	(0.05; 0.121)	0.770	98	*Q* = 11.69; df = 1 *p* = 0.0029
	(> 2015)	14	9.02%	(0.061; 0.146)	0.86	97	
Cystic echinococcosis							
Region-wise	SNNPR	7	22%	0.1359; 0.3278	0.5679	97	*Q* = 6.67; df = 4 *p* = 0.023
	Oromia	12	33%	(0.2245; 0.4531)	0.8474	98	
	Tigray	5	29%	(0.2434; 0.3338)	0.0565	97	
	AA	5	14%	(0.0649; 0.2773)	0.9401	97	
	Amhara	8	23%	(0.1491; 0.3428)	0.6043	98	
Year-wise	2008–2013	13	22%	(0.1480; 0.3185)	0.8172	98	*Q* = 0.66; df = 2 *p* = 0.7206
	2014–2016	14	26%	(0.1891; 0.3370)	0.5374	97	
	2017–2023	10	28%	(0.1756; 0.4137)	0.9190	98	

### Estimated proportions of cystic echinococcosis and *Cysticercos bovis* found in the same organ

In this study, *C. bovis* and hydatid cysts were identified as the most important foodborne parasites at post-mortem examination at the slaughterhouse. The main organs affected by these parasites include the lungs, heart, liver, kidneys, and shoulder and masseter muscles. The included studies were categorized as (i) articles examining both parasites simultaneously in the same organ, (ii) studies treating only *C. bovis* in a single organ, and (iii) articles addressing only the distribution of cystic echinococcosis in a single organ (kidney).

### Simultaneous distribution of *Cysticercos bovis* and cystic echinococcosis in the lungs, heart, and liver

**Figure 18:**
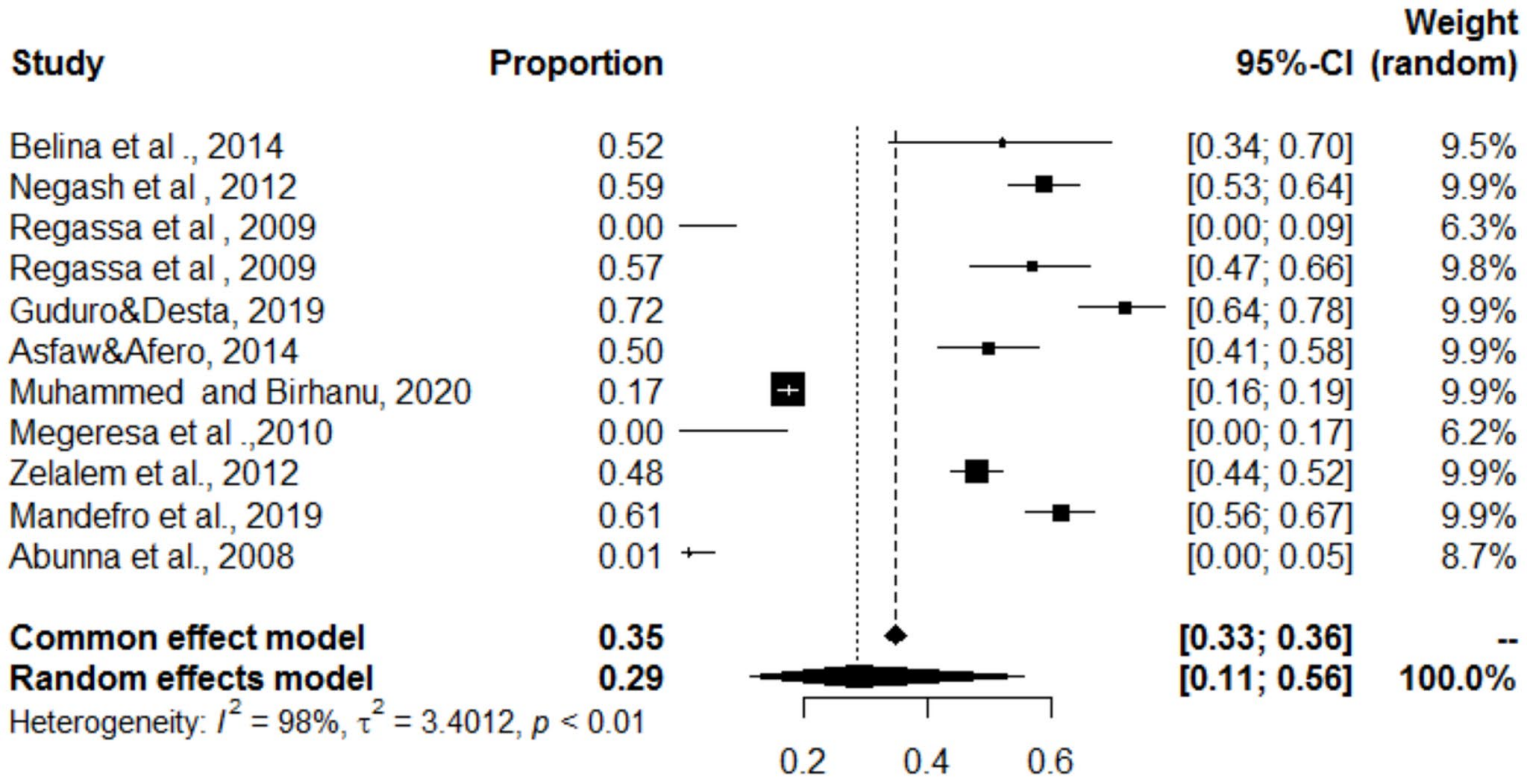
Pooled prevalence of *C. bovis* and cystic echinococcosis in the lungs.

**Figure 19:**
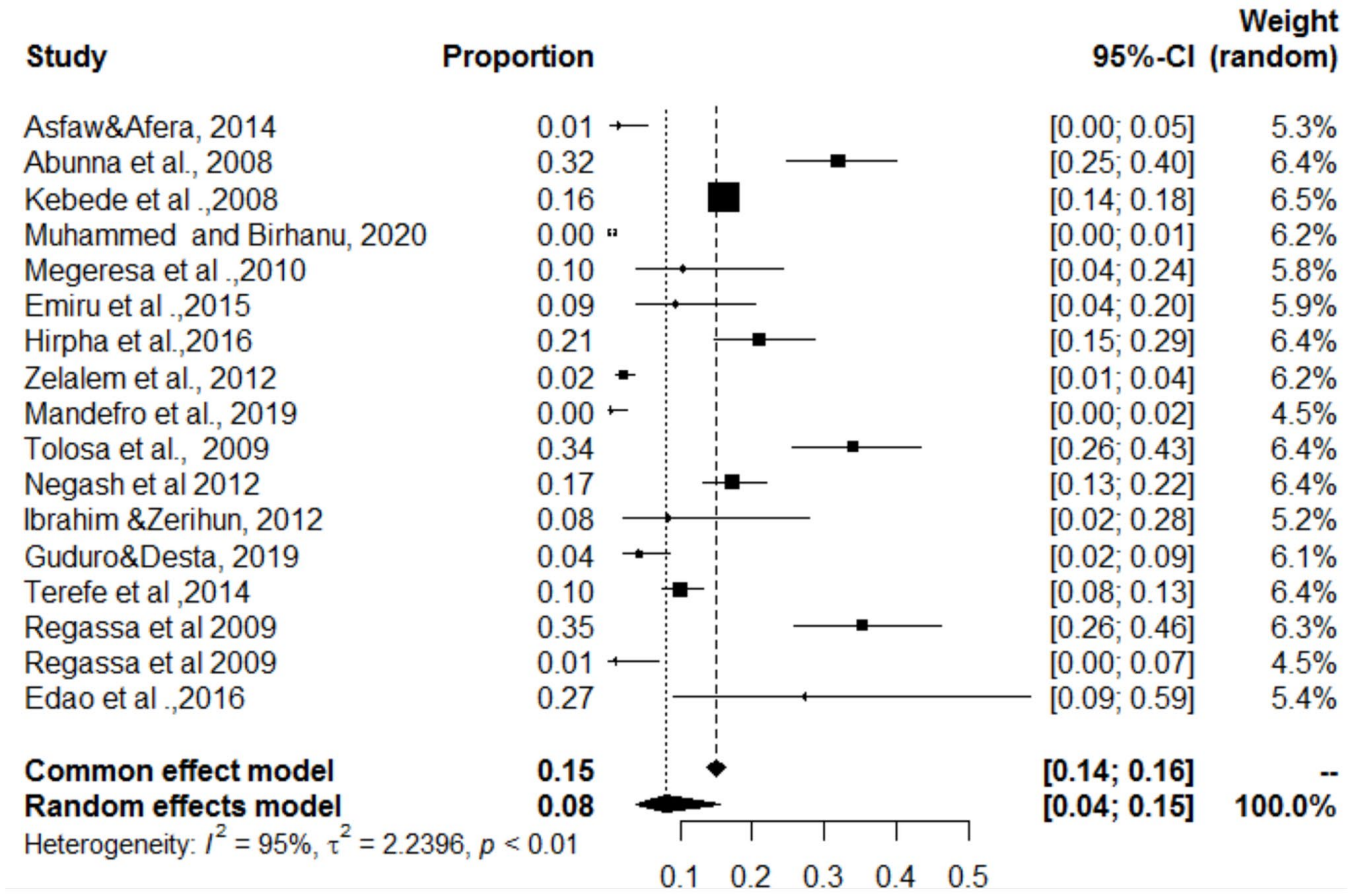
Pooled prevalence of bovine cysticercosis and cystic echinococcosis in the heart.

**Figure 20:**
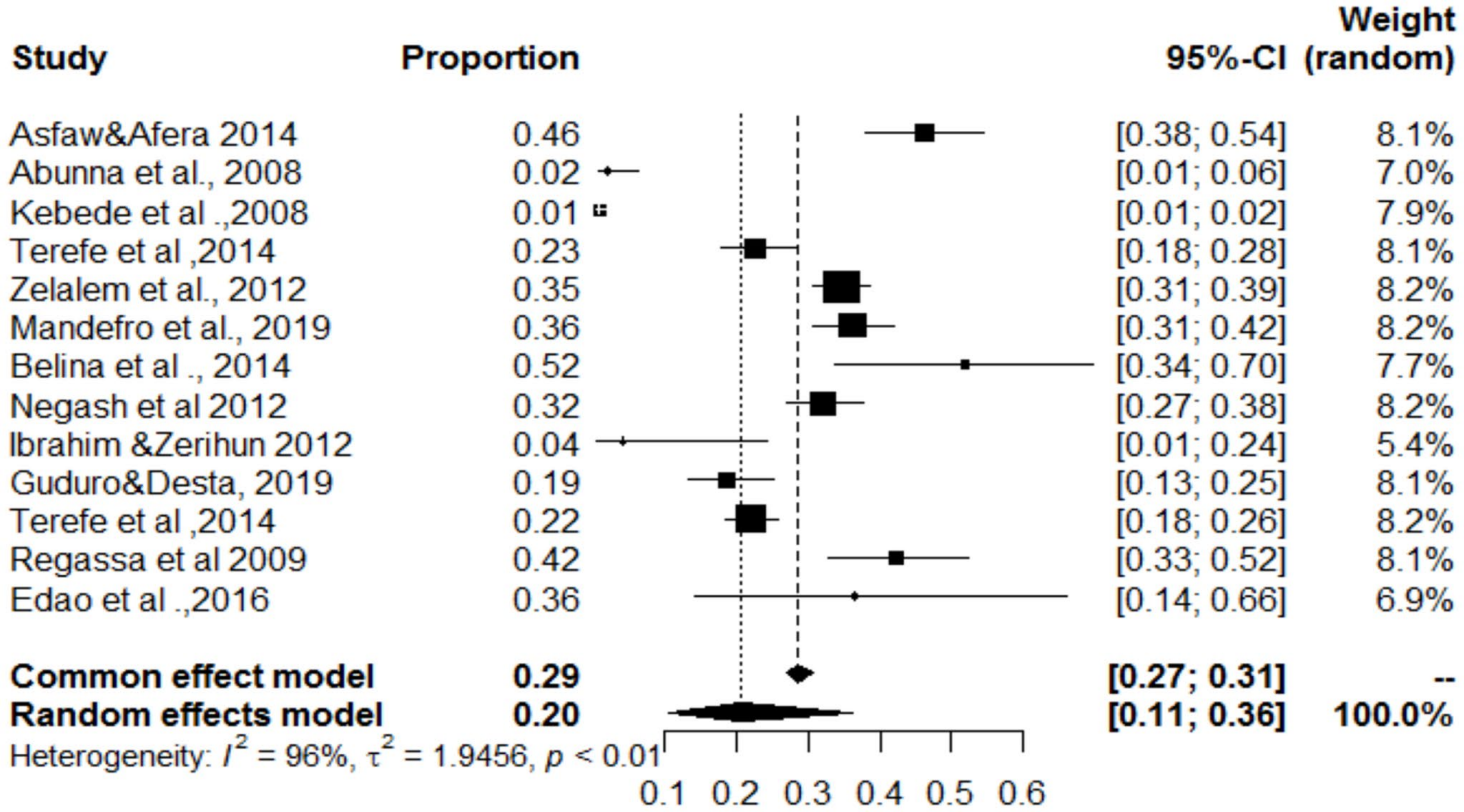
Pooled prevalence of bovine cysticercosis and cystic echinococcosis in the liver.

In the present study, the lungs, heart and liver were affected by both *C. bovis* and cystic echinococcosis (same study). The overall pooled prevalence rates of both *C. bovis* co-infections with cystic echinococcosis of the lungs, liver and heart were 29, 20 and 8%, respectively (Figures 18–20). In the case of the lung, as shown in Table 6, a subanalysis was performed on the basis of the categorization of studies under *C. bovis* (eight articles) and cystic echinococcosis (only three articles), and the pooled prevalence was found to be 51 and 1%, respectively. Similarly, subanalyses of the included studies were based on parasite type, and the pooled prevalence rates of *C. bovis* and hepatic hydatosis were 8 and 36%, respectively (Table 6). In addition, subgroup analysis of studies based on parasite type revealed that the pooled prevalence of *C. bovie* with cystic echinococcosis in the heart was 14 and 3%, respectively (Table 6).

### Overall estimation of the distribution of cystic echinococcosis in the kidney

In our study, the kidney was the only organ affected only by cystic echinococcosis. Among a total of 37 studies, six reported infection of the kidney by cystic echinococcosis. The pooled proportion of cystic echinococcosis in the kidney was 2% (95% CI; 1–6%), with high inter-study heterogeneity (*I*^2^ = 96%; *p* < 0.01) (Supplementary material 3).

### Overall distribution of *Cysticercos bovis* alone in different organs at post-mortem inspection

In this meta-analysis, the highest levels of *C. bovis* were observed in the shoulder muscle, and the tongue was the second most susceptible organ. Regarding the anatomical distribution of the cysts, 32% of the affected organs were located in the shoulder region, with minimal heterogeneity observed in this proportion (Cochran’s *Q* = 11.21 and *p* value of 0.0474, *I*^2^ statistic of 55%). The deltoid, 21% (*I*^2^ = 83%; *Q* = 53.32, *p* = 0.000) of the tongue, 19% (*I*^2^ = 66.3%, *Q* = 26, 72, *p* = 0.0016) and 6% (*I*^2^ = 86.3%, *Q* = 46.52, *p* = 0.0001) of the tongue (Supplementary materials 4a–d). Among the organs examined, the diaphragm was least affected by *C. bovis*, as summarized in Table 8.

**Table 8:** Summary of parasite zoonotic distributions in different organs at post-mortem inspection.

Parasite	Organ	PP (%)	*N*	95%CI	*I* ^2^	tau^2^	Test of heterogeneity
							Q, d.f., and *P* value
Hydatosis and *C. bovis*	Lung	29	11	(11–56%)	98	3.4	
Hydatosis and *C. bovis*	Liver	20	13	(11–36%)	96	1.94	282.80, 12, and <0.0001
Hydatosis and *C. bovis*	Heart	8	17	(4–15%)	95	2.23	340.1, 16, and <0.0001
*C. bovis*	Shoulder	32	6	(27–37%)	55	0.039	11.21, 5, and 0.0474
*C. bovis*	Masseter	19	10	(16–22%)	66	0.045	26.72, 9, and 0.0016
*C. bovis*	Tongue	21	10	(15–30%)	83	0.43	53.32, 9, and 0.000
*C. bovis*	Diaphragm	6	6	(3–14%)	98	0.98	46.5, 5, and 0.0001
*C. bovis*	Lung	1	11* (2)	(0–4%)	0	0	629.82, 1, and <0.0001
Hydatosis	Lung	51		(38–64%)	99	0.54	
*C. bovis*	Liver	8	13* (2)	(2–26%)	97	2.58	6.62, 1, and 0.0101
Hydatosis	Liver	36		(29–44%)	82	0.17	
*C. bovis*	Heart	14	17* (2)	(7–26%)	96	1.76	6.35, 1, and 0.0117
Hydatosis	Heart	3		(1–8%)	93	1.6	
Hydatosis	Kidney	2	6	(1–6%)	96	1.46	

## Discussion

The present systematic review aimed to assess the prevalence of common animal-origin foodborne parasitic zoonoses in the meat of domestic ruminants in Ethiopia from 2008 to 2023. Our meta-analysis reveals a concerning picture, with pooled prevalence estimates of 38% for toxoplasmosis, 25% for cystic echinococcosis, 14% for cryptosporidiosis, and 9% for bovine cysticercosis. These figures underscore the significant public health and economic burdens imposed by these infections on Ethiopian livestock production. The highest pooled prevalence was for toxoplasmosis (38%), a finding that is likely linked to the limited availability of specific treatments for *Toxoplasma gondii* in animals, as well as the frequent cohabitation of small ruminants with cats, the parasite’s definitive host, especially in rural areas where most abattoir meat originates. It’s worth noting that this pooled prevalence falls within the range reported in previous Ethiopian studies, being lower than some (57, 74.9, 70.5, 43.1%) and higher than others (31, 33, 19.7%), which can be attributed to variation in serological tests employed, intermediate/definitive host relationship, sample size used, and geography. As reported by Woldesenbet and Harito (56), *Toxoplasma gondii* is a significant public health concern in Ethiopia, contributing to the high pooled prevalence observed in this meta-analysis. Almuzaini (57) emphasizes the role of food chain contamination in the widespread prevalence of zoonotic toxoplasmosis. The incidence rate of toxoplasmosis in Ethiopia exceeds those reported in some other African countries like Somalia (15.9%) and Sudan (32%), which points to the effect of sociocultural practices, husbandry methods, and public awareness. Despite the increasing knowledge and awareness of the disease there is still an increase in outbreaks because of poor and improper food handling. The need for further DNA vaccines is supported by Chen et al. (58). Dubey et al. (59) recently updated the worldwide rate of congenital toxoplasmosis in humans.

In terms of species, the pooled seroprevalence was highest in sheep (41%), followed by goats (39%) and cattle (28%). Factors contributing to the observed differences include variations in environmental factors, cat density, study design, management practices, diagnostic methods, host species, animal status, age, breed, serological tests, parasite/host genetics, immune response, cultural practices, and feeding habits. While our finding for goats (39%) aligns with some prior reports from Northwest and Southwest Ethiopia, it differs from others, underscoring the regional variation in *Toxoplasma gondii* seroprevalence. Hussein et al. (60) identification of Sarcocystis species in sheep and goats shows a clear connection with foodborne parasitic zoonoses. The seroprevalence of *T. gondii* in sheep (41%) was similar to findings from a study that reported a 37.9% seroprevalence in Central Ethiopia (61). The variation in seroprevalence between countries and continents is possibly due to wild animal contact and environmental conditions which influence the epidemiology of toxoplasmosis. Prevention in accessing pastures to grazing areas reduces the epidemiology of toxoplasmosis.

Cattle exhibited a lower pooled seroprevalence (28%) compared to small ruminants, possibly due to reduced contact with cats, with sheep and goats being often raised at higher altitudes, increasing infection risk. Diagnostic methods also played a role, with ELISA yielding the lowest (23%) and LAT the highest (58%) prevalence rates, due to technical factors and inherent test characteristics. Overall, the prevalence of toxoplasmosis in our study exceeds that reported in a molecular meta-analysis worldwide (14.7% in sheep tissues), which is possibly due to meat consumption methods and the slaughter system used.

Our meta-analysis also found an overall pooled prevalence of *Cryptosporidium* infection of 14% among commonly consumed domestic red meat animals. This aligns with a previous report from Bishoftu, Ethiopia (14%), but contrasts with findings from Egypt (20%). It’s worth noting that molecular characterization of *Cryptosporidium* is increasingly refining our understanding of transmission (62–65). Given the importance of this parasite especially in the immunocompromised population, a review of the global burden of Cryptosporidium highlights the ongoing need for improved diagnostics and treatments as recorded by Javed and Alkheraije (66). Giardia and Cryptosporidium spp. are frequently detected together in domestic animals. The difference in animal husbandry and grazing practice may have influenced the exposure and occurrence rates as mentioned by Megersa et al. (67). Stocking rates and husbandry systems are the major contributors to the variations in these countries.

Our analysis revealed higher infection rates in young animals (15%), suggesting that cryptosporidiosis poses a greater risk among calves, lambs, and kids in Ethiopia, this may be associated with colostrum related hygienic and diarrheic conditions. These findings align with numerous prior reports. Lower infection rates were reported in Iran at 4.7% (68), China at 5.09% (69), and Ethiopia at 13% (20). However higher prevalence rates were reported in various countries. Local climatic situations, strategic deworming, sample size, sampling method, study regions, diagnostic methods, feeding habits, environmental/farm hygiene, season, livestock management (production system), colostrum-feeding practices, herd size, composition and breed, confection and levels of close contact contributed to the differences of Cryptosporidium.

For cystic echinococcosis parasites, the overall pooled prevalence was 25%, which was comparable with that reported by Tkubet et al. (70) in the Tigray regional state. A number of factors contribute to this high prevalence of the parasite as reported by Dima and Jemal (71). Epidemiological characteristics and risk factors for cystic echinococcosis have been analyzed in national population-based field surveys (72). Recent studies highlight the ongoing presence of cystic echinococcosis (158) and emphasize the importance of understanding liver cystic echinococcosis. Abdulla (73), notes that the socioeconomic and habit of proper handling of slaughterhouse animals are important factors that influence the variations in prevalence. The variation in prevalence in different regions or localities might be due to differences in agro-ecologic situations. The serology for diagnosing human hepatic cystic echinococcosis and its relation with cyst staging has been reviewed (74).

In the present meta-analysis, the pooled prevalence of bovine cysticercosis was recorded (9%). There are various implementations in the rigorous implementation of meat inspections as noted by Netsanet et al. (75). The need for improved sanitation measures and proper animal hygiene practices should be implemented. The rigor of implementation of meat inspection measures, expansion of public health standards, and various hygienic practices resulted in a higher prevalence than 0.2 and 3% in South Africa and Rwanda, respectively. Mathewos et al. (76) note that the economic significance of the slaughterhouse and handling are important for the economic loss of the bovine.

While Qamar and Alkheraije (77) focused on *Haemonchus contortus* resistance, and Rehman et al. (78) provide a review of vaccine development against *Fasciola* it is important to take into considerations that the study also promotes vaccine formulation for other foodborne parasites and the impact of foodborne parasites on public awareness. Kebede et al. (79) noted the importance of risk factors in cattle trematodiasis and how it effects the trematode infection. Netsanet et al. (75) conducted studies on the effects of deltoid location followed by the tongue on masseter in varying degree of location and geographical and environmental conditions and blood kinetics. Organized and strict meat inspection practices in abattoirs and in-depth awareness of society can ensure that meat is free from high levels of metacestode infection. Overall, this investigation revealed that cystic echinococcosis and toxoplasmosis are the most prevalent zoonotic parasites. The control of these parasitic infections is not just an animal health issue; it’s also a public health and economic imperative for Ethiopia. Addressing these challenges requires a multifaceted approach encompassing improved sanitation, better animal husbandry practices, strategic anthelmintic use, and comprehensive public health education.

## Limitations

Although this study has certain limitations, it provides significant epidemiological data on the prevalence and spread of endemic foodborne parasitic zoonosis in Ethiopia, which will be useful for disease prevention and control. First, there was an uneven distribution of studies across regions, hosts, study periods, study types, and sample sizes; this shows that the results may not accurately reflect the situation for Ethiopia. The second constraint is that cattle, sheep and goats can only be labeled or consumed by humans according to Ethiopian norms, and the third constraint could be derived from Ethiopian norms, e.g., Dogs, cats, horses, donkeys, mules and others that may not be consumed by Ethiopia do not help to draw representative results. Another important limitation of this review may be that the selected parasites do not represent all zoonotic parasitic diseases of animal origin. In addition, the diagnostic procedures and the sources of the data/samples may also introduce bias and may not represent the accurate distribution of the zoonotic foodborne parasite of animal origin, as most of the samples were collected at the slaughterhouse.

## Conclusion

This meta-analysis indicated that the overall pooled prevalence of bovine cysticercosis and cystic echinococcosis among red meat sources from domestic ruminants in Ethiopia has increased in recent years. The subgroup analysis reveals a high overall pooled prevalence of toxoplasmosis in ovine species and cryptosporidiosis in cattle. These findings are expected to enhance on-going efforts to address food security in Ethiopia and support the implementation of food and nutrition security control strategies. The results emphasize the need for critical attention to communities with habits of consuming partially cooked or raw meat. Additionally, this study provides valuable insights that could shape future research and policy decisions related to food safety, particularly concerning the origin of animal-based foods. To address these public health and economic impacts effectively, multisectoral collaboration involving public health scientists, ecologists, veterinarians, economists, and other relevant stakeholders is essential. Furthermore, it is important to plan and implement joint in-service training programs for targeted workers across various sectors.

## Data Availability

The original contributions presented in the study are included in the article/Supplementary material, further inquiries can be directed to the corresponding author.
